# Solar Energy Storage by Molecular Norbornadiene–Quadricyclane Photoswitches: Polymer Film Devices

**DOI:** 10.1002/advs.201900367

**Published:** 2019-04-25

**Authors:** Anne Ugleholdt Petersen, Anna I. Hofmann, Méritxell Fillols, Mads Mansø, Martyn Jevric, Zhihang Wang, Christopher J. Sumby, Christian Müller, Kasper Moth‐Poulsen

**Affiliations:** ^1^ Department of Chemistry and Chemical Engineering Chalmers University of Technology SE‐412 96 Gothenburg Sweden; ^2^ Department of Chemistry University of Copenhagen Universitetsparken 5 2100 Copenhagen Ø Denmark; ^3^ Department of Chemistry The University of Adelaide Adelaide SA 5005 Australia

**Keywords:** heat release, solar energy storage, solar thermal, solid state materials

## Abstract

Devices that can capture and convert sunlight into stored chemical energy are attractive candidates for future energy technologies. A general challenge is to combine efficient solar energy capture with high energy densities and energy storage time into a processable composite for device application. Here, norbornadiene (NBD)–quadricyclane (QC) molecular photoswitches are embedded into polymer matrices, with possible applications in energy storing coatings. The NBD–QC photoswitches that are capable of absorbing sunlight with estimated solar energy storage efficiencies of up to 3.8% combined with attractive energy storage densities of up to 0.48 MJ kg^−1^. The combination of donor and acceptor units leads to an improved solar spectrum match with an onset of absorption of up to 529 nm and a lifetime (*t*_1/2_) of up to 10 months. The NBD–QC systems with properties matched to a daily energy storage cycle are further investigated in the solid state by embedding the molecules into a series of polymer matrices revealing that polystyrene is the preferred choice of matrix. These polymer devices, which can absorb sunlight and over a daily cycle release the energy as heat, are investigated for their cyclability, showing multicycle reusability with limited degradation that might allow them to be applied as window laminates.

## 1 Introduction

One of the main challenges in the world today is a sustainable energy production. In 2017, 85% of world energy production was fossil fuel derived,^1^ and environmental impacts necessitates the global community to seek cleaner alternatives.^2^ Renewable green energies derived from solar power, wind, or hydroelectric sources are the most commonly implemented. However, daily and seasonal variations in both energy production and consumption compel the development of new energy storage technologies. One solution is to address both light harnessing and storage in a combined technology. This has been shown in molecular solar thermal (MOST) storage^3^ also referred to as solar thermal fuel (STF).^4^ Here, energy can be stored in a molecular photoswitch through irradiation under sunlight, resulting in the formation of a high energy isomer. This metastable species can be stored until the energy is released as heat, leading to the reformation of the parent form. Such heat release can be prompted by heating or even occur under ambient conditions depending upon the storage life time (*t*_1/2_) of the high‐energy isomer. Moreover, this process has been shown to occur through a stimulus such as a heterogeneous catalyst,^5^ electric potential,^6^ or light.^7^ Molecular photoswitch systems that have been studied for MOST include azobenzenes,^4^, ^8^ tetracarbonylfulvalenediruthenium complexes,^3^, ^9^ dihydroazulenes,^10^ and NBDs.^11^

A promising use for liquid MOST systems is in heating applications,^12^ whereas the application of such systems in the solid state could lead to functional windows for climate control and de‐icing,[qv: 8f,13] thus reducing the energy need for conventional heating and cooling. However, in order to make such coating applications practically viable, there are some requirements for the molecular photoswitch system that must be fulfilled.[qv: 11a] These include molecular properties such as a good overlap between the absorbance spectra of the parent molecule with the solar spectrum, where the high‐energy metastable isomer should not exhibit a competing absorbance; good quantum yield for the photoisomerization; high energy density, and good cyclability. Lastly, the storage time of the metastable isomer should fit the desired application. For instance, this could be as short as 4–8 hours for a lamination on windows that regulates the interior temperature on a daily basis. On the other hand, long‐lived liquid based metastable compounds have a possible preferred application in catalyst controlled heat release for domestic heating in extreme seasonal climates. The coating, which could conceivably be the neat molecular photoswitch as a solid or a polymer‐based composite, needs to retain the desirable molecular properties of the photoswitch, display excellent cyclability, energy density, and stability, and demonstrate ideal aesthetics in, for instance, window laminate applications.

**Figure 1:**
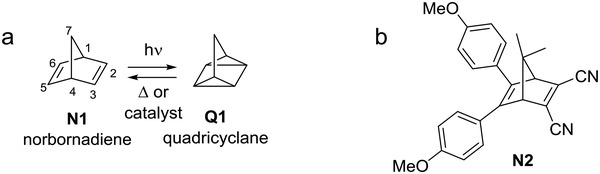
a) Norbornadiene (NBD) **N1**–quadricyclane (QC) **Q1** system with numbering scheme for NBD. b) NBD derivative **N2** with C2/C3 cyano acceptors and C5/C6 donor units previously studied in PMMA.[qv: 14a]

One molecular system that has shown promise in this regard is the norbornadiene–quadricyclane (NBD–QC) couple (**Figure** 1). The photoswitching properties are highly tunable for MOST, in fact, many derivatives have been shown to exhibit good cyclability and high quantum yields.[qv: 11f] Unfortunately, the absorbance band of unsubstituted **N1** does not overlap with the solar spectrum. By introducing a π‐conjugated donor–acceptor system across C2 and C3, the absorbance of the NBD can be red‐shifted.[qv: 5f,11e,g‐i] However, a higher degree of polarization through the C2–C3 olefin is typically accompanied by shorter ambient lifetimes for the corresponding QCs.[qv: 11h] We have recently shown that this effect can be counterbalanced with subtle structural modifications to the aromatic group at the C3 position of NBD, which allowed significant increases of the storage time (*t*_1/2_) by a factor of 100, without compromising the energy release (Δ*H*_storage_) or the absorbance profile.[qv: 11h] Furthermore, dimeric molecules having a linear conjugation pathway between the two NBD units through a shared benzene donor display both good solar spectrum overlap and quantum yields.[qv: 11g] In addition, the concept of sharing the donor part of the molecule between several NBD units effectively leads to a lower molecular weight per NBD unit and in consequence to a higher energy density; for the double metastable form approaching up to ≈0.93 MJ kg^−1^.[qv: 11g]

In an effort to realize solid state devices for application, the MOST photoswitch will ideally be incorporated into polymers. To this end, Zhitomirsky and Grossman[qv: 8e] designed high molecular weight azobenzene derivatives that were able to form solid films, retaining morphology through interconversion processes. In addition, methyl methacrylate‐functionalized azobenzene afforded a polymer with high cyclability, with a half‐life of longer than two days.[qv: 8d] NBD derivatives have also been incorporated into polymers, either by incorporating it into a poly(methyl methacrylate) (PMMA) host^14^ or by making it part of the polymer itself.^15^ In addition, many NBDs, or their corresponding QCs, are typically liquids. Thus, blending with a polymer makes it possible to create a solid photoactive NBD material, whose mechanical properties are governed by the polymer matrix in which the NBD has been dispersed. Another advantage of using NBDs for this purpose is that the QC form tends not to absorb visible light, thereby avoiding inner filtering effects or photostationary states, which is more evident for other photochromic systems. Good cyclability has been demonstrated for **N2** (Figure 1) hosted in PMMA, though the reported half‐life was less than 5 min, which has a limited practical application.[qv: 14a]

The focus of the work presented here is to develop an NBD–QC polymer composite system toward applications in windows and coatings where solid‐state function is needed. While the NBD system fulfils many of the requirements stated above for molecular entity, few examples of function in the solid state can be found for this system, likely due to the challenge of balancing energy storage time with solar spectrum match.[qv: 11g,h] Here, we present the synthesis of a new series of NBD‐based molecules with a good solar spectrum match (estimated up to 3.8% solar energy storage efficiency), using the strong acceptor moiety trifluoroacetyl unit in conjunction with carefully selected donor units for the purpose of making NBD derivatives having QCs with half‐lives in the range of 4–8 h, applicable for a daily charging/discharging cycle. Furthermore, we investigated the performance of two high‐performing derivatives in film configuration for different polymer matrices demonstrating the basic function for a possible future application in MOST coated windows.

## 2 Results and Discussion

### 2.1 Synthesis

**Scheme 1:**
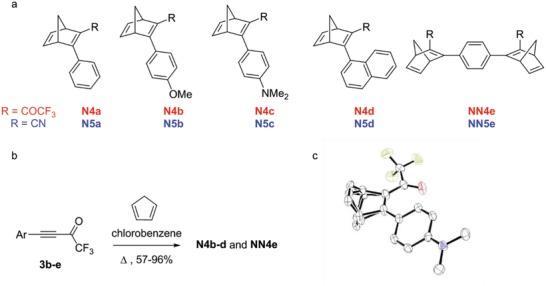
a) Previously studied NBDs (**N5a–d**, **NN5e**, and **N4a**) and compounds made for this study (**N4b–d** and **NN4e**). b) Reaction protocol used to form **N4b–d** and **NN4e**. c) A representation of the structure of **N4c** obtained by X‐ray crystallography on crystals grown from dichloromethane/*n*‐heptane. The disorder of the NBD moiety (major/minor 0.59:0.41) is shown but hydrogen atoms are omitted. The conformation obtained by solving the crystal structure highlights that the C_carbonyl_‐C2_NBD_‐C3_NBD_‐C_aryl_ dihedral angle is 11.5° for the major form, indicating that the aryl and trifluoroacetyl groups are nearly coplanar.

With a target of MOST window laminates possessing a daily charging/discharging cycle in mind, we designed a series of NBD‐based molecules which should possess a good solar spectrum match and target half‐lives in the range of 4–8 h. **Scheme** 1 shows the structures of previously studied NBDs with the cyano acceptor **N5a–d** and **NN5e** and parent NBD with the trifluoroacetyl acceptor moiety **N4a**. Based on our previous studies,[qv: 11h,i] we chose to investigate the properties of better aromatic donors used in conjunction with the strong trifluoroacetyl acceptor **N4b–d** and the double NBD analogs **NN4e**. These NBD derivatives were made using a Diels–Alder methodology between a selection of trifluoroacetylphenyl acetylenes with cyclopentadiene. The acetylenic precursors **3b–e** were made following a literature procedure from their corresponding terminal alkynes.^16^ Dienophiles **3b–e** could be heated in the presence of cyclopentadiene to form **N4b–d** and **NN4e** in excellent yields (Scheme 1), primarily due to the strong trifluoroacetyl acceptor activating the alkyne toward the [4+2π] cycloaddition. The potential for extended conjugation of the C2–C3 substituents was confirmed through a single crystal X‐ray structure for **N4c**, which revealed near coplanarity of these substituents, although there is some disorder between the bridge C7 and the double bond of C5–C6 (Figure 3).

### 2.2 Photochemical Properties

To determine whether the newly synthesized NBD–QC photoswitches had the correct physical properties for polymer incorporation and daily cycle laminate applications, all NBDs were studied by UV–vis spectroscopy in toluene (**Table** 1). This also included measuring the quantum yield of photoconversion and kinetic stabilities all QCs. Comparing the previously made NBDs **N5b–d** bearing a cyano acceptor group to analogs **N4b–d** with trifluoroacetyl acceptor group, the latter experienced red‐shifting for the NBD absorbance spectra of 70–100 nm. This gave NBD products with an improved overlap with the most intense region of the solar spectrum, especially **N4c**, which had an onset of absorbance at 529 nm. Meanwhile, there appeared to be no observable trend between the quantum yields for photoconversion between series **N4b–d** and **N5b–d** when measured using the high concentration regime (Abs >2).^17^ Using the molecular absorptivity, quantum yield of photoisomerization and energy storage density (Δ*H*_storage_) (see below) of the isomers as input parameters, we can estimate the fraction of the solar spectrum that can be captured and stored by these compounds. The estimates reveal energy capture efficiencies of 2.9%, 1.5%, and 3.8% for **N4b**, **N4d**, and **NN4e** respectively, (see the Supporting Information for details) rendering these the series of compounds with best solar capture efficiencies reported from our group so far.

**Table 1:** Properties of NBDs made in this study featuring absorbance, quantum yield, and kinetic data measured in toluene compared against previously reported NBDs

	λ_max_ [nm] [ε × 10^3^]	λ_onset_ ^a)^ [nm]	QC λ_onset_ [nm]	Φ/photo conversion [%]	*t*_½_
**N5a**[qv: 11e]	309 (7.7)	358	–	58	55 days
**N4a**[qv: 11i]	323 (5.2)	426	–	53	72 h
**N5b**[qv: 5f]	326 (13.3)	380	–	61	30 days
**N4b**	374 (8.2)	457	402	68	6.6 h
**N5c**[qv: 11h]	374 (6.5)	427	401	73	7 h
**N4c**	450 (18)	529	488	54	0.64 h
**N5d**[qv: 11h]	321 (7.6)	373	325	59	18 years
**N4d**	354 (3.4)	439	341	46	10 months
**NN5e**[qv: 11g]	350 (23.6) (NN)	400 (NN)	252(QQ)	73^c)^ 51^d)^	11 days(QQ) 2 days (NQ)
**NN4e**	386 (12) (NN) 355 (QN)^b)^	466 (NN) 450 (QN)^b)^	417 (QQ)	77	17 h (QQ) 3 days (NQ)

^a)^ Absorption onset defined as log *e* = 2

^b)^ Estimated from Figure 2b, as pure **QN4e** was not formed

^c)^ For the first conversion from NBD–NBD to QC–NBD

^d)^ For the second conversion from QC–NBD to QC–QC.

**Figure 2:**
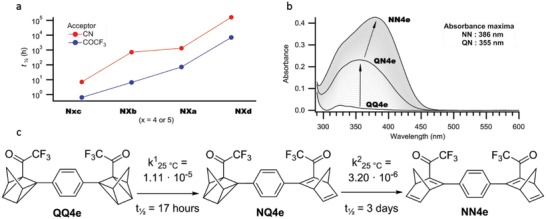
a) The evolution curve of half‐life and acceptor strength between **N5a–d** (red) and **N7a–d** (blue). b) Absorbance spectra of the progressive back‐conversion reaction of **QQ4e→NQ4e→NN4e** in toluene. c) Scheme showing the sequential back‐reaction steps observed in the UV–vis spectrum for **QQ4e** to **NN4e**.

The kinetic decay for all QCs was measured at three different temperatures and from exponential fits, the rate constants and life‐times were obtained. From Arrhenius plots (see the Supporting Information), the room temperature life‐times were determined in toluene by extrapolation. Not surprisingly, the larger the dipole moment through the C2–C3 olefin for the NBD, the lower the life‐time for the corresponding QC forms. Metastable forms **Q4b** and **Q4c** exhibited markedly shorter half‐lives than **Q4a**, in the order of a few days for previously reported **Q4a** to around 7 h for **Q4b** and less than an 1 h for **Q4c**. In fact, parallel trends for the half‐lives of **Q5a–d** with a cyano group as the acceptor were noticed. Exchanging the cyano for trifluoroacetyl unit proportionately afforded an ≈20‐fold increase in the back‐conversion rate to the corresponding NBD (**Figure** 2a). Importantly, the effect of having a substituent at the *ortho* position was evident in the trifluoroacetyl acceptor series, stabilizing the QC and showing a similar trend in previously reported for **Q5d** and other 2‐cyano‐3‐arylNBDs.[qv: 11h]

As observed for **N4b–d** the dimer **NN4e** also exhibited a red‐shifted onset for the absorbance relative to **NN5e**. Previously synthesized **NN5e** displayed stepwise photoconversions by irradiation at 405 nm, where an **NQ5e** intermediate could be identified.[qv: 11g] In comparison, **NN4e** did not experience any sequential photoswitching events. However, stepwise kinetics did occur for the back‐conversion for **QQ4e**. It was found that the first conversion (**QQ4e→NQ4e**) was significantly faster than the second step (**NQ4e→NN4e**) (Figure 2c), which is surprising since it is opposite to what we observed for previously explored double systems.[qv: 11g] This could be clearly observed from the progressive change in the maxima for the absorbance profile during a kinetic experiment. As seen in Figure 2b, the absorbance maximum initially remains unchanged, where the conversion of **QQ4e** to **NQ4e** had occurred relatively fast, and thereafter the maximum started to slowly red‐shift, a result of **NQ4e** to **NN4e**. Indeed, this progressive behavior was substantiated by following the back‐conversion by NMR (see the Supporting Information). Two different half‐lives were also observed for dimer **NN5e**, though in this case the conversion for **NQ5e** to **NN5e** was found to be faster than the conversion from **QQ5e** to **NQ5e**.

The quantum yields for the two processes in this photoconversion were examined at both 340 and 405 nm, where the former wavelength was close to the absorption maximum for **NQ4e** and the latter favored the absorbance of the **NN4e**. At both these wavelengths, comparable results were obtained, both showing a linear fit for the change in concentration versus irradiation time, and so the quantum yields for both processes were 77%. The light‐harvesting properties for **NN4e** were superior to **NN5e**, which had sequential quantum yields of 73% for the first photoconversion (**QQ5e→NQ5e**) and 51% for the second event (**NQ5e→NN5e**).

### 2.3 Incorporation of NBDs into Polymers

With the new NBD–QC photoswitches and analysis of the photochemistry in hand, the study focused on the incorporation of the identified NBDs into a polymer matrix to assess possible applications in climate control for window laminating. Ideally, laminated films for temperature regulation require the NBD to have a half‐life in the order of 4–8 h. To investigate this, **N4b** was selected as it has a good solar spectrum overlap (λ_onset_ = 457 nm) and a good quantum yield (Φ = 68%), but most importantly a half‐life of just under 7 h in toluene. This photoswitch was incorporated into four different polymers with the aim of investigating the effect of the matrix polymer on the switching properties of the incorporated NBD. This included the use of atactic polystyrene (PS), which was identified as a comparable host medium to solution based studies in toluene. Also, included in this study was atactic PMMA as it has been reported previously that the incorporation of NBD derivatives into PMMA resulted in composites with good cyclability. To compliment this investigation, more polar polymer matrices such as polycarbonate (PC) and polyvinylidene chloride (PVDC) were also tested. PVDC was chosen as it provides a good barrier for molecular oxygen, and it was evident from former work that molecular oxygen can affect the stability of NBD undergoing several cycles.[qv: 11f] Each polymer film was prepared by allowing a dichloromethane solution containing 0.1 wt% **N4b**. All composites of **N4b**@polymer gave free standing films of yellow‐colored plastic, and when subjected to irradiation these materials went colorless, through the formation of **Q4b**.

**Figure 3:**
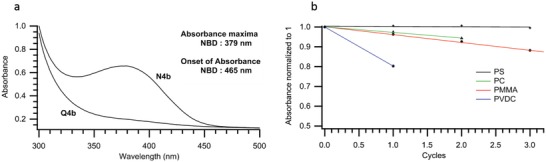
a) Absorbance spectra of 0.1 wt% **N4b**@PS before and after irradiation. b) Cyclability of 0.1 wt% **N4b** in different polymers.

The absorbance profile for **N4b** within the polymer matrices were slightly redshifted compared to solution characterization in toluene (order of 3 nm). **Figure** 3a shows the UV–vis absorption profile found for **N4b**@PS before and after irradiation. In addition, cycling studies were performed on these films, and the results for the activity of **N4b** in the different hosts can be seen in Figure 3b. Each cycle consisted of measuring absorbance followed by irradiation of the sample until the color of the film had faded and the peak at 379 nm in the UV–vis absorption spectrum had disappeared. The sample was then left in the dark, and the UV–vis absorption spectrum was periodically measured until no change in the spectra was observed.

The stability of **N4b** was indeed dependent upon the host polymer. For instance, composite **N4b**@PVDC after one cycle had lost some 20% of its activity, whereas **N4b**@PS formed a much more robust film, experiencing very little degradation after four cycles.

The half‐lives of **Q4b** in PMMA, PC, and PS at room temperature were carried out and summarized in **Table** 2. It was found that the rate of back‐conversion for **Q4b→N4b** was significantly influenced by the polymer host. Composite **Q4b**@PMMA exhibited a fast rate of conversion with a half‐life of only 20 min, while for **Q4b**@PS and **Q4b**@PC, the photoswitch exhibited markedly longer half‐lives. In fact for **Q4b**@PS and **Q4b**@PC, studies of the switching kinetics revealed that the back‐conversion did not follow a first order kinetics, and fitting of the absorbance as a function of time instead agreed with a double exponential decay. For instance, **Q4b**@PS exhibited two half‐life times of 40 min and of 5.8 h for the second cycle. Similar results for other photoswitches in polymer films have been reported[qv: 10c] and could be explained by the inhomogeneous nature of local environment in the polymer composites. From these results, we can determine that polystyrene was the most suitable matrix polymer for the fabrication of **N4b**‐polymer composites on the account of its high durability and a high lifetime stability for the QC form. In addition, this aggregation does not appear to affect the stability of the photoswitch in PS. This makes **N4b**@PS a promising material for window tinting applications. However, this is complicated by the changing rates after successive cycles. Due to the low stability of this photoswitch in PVDC, no attempt was made to analyze the back‐conversion kinetics for this material.

**Table 2:** Results of kinetics measurements of polymer films, featuring the rate constants as 1 and 2 for the double exponential fit and corresponding half lives

	Cycles	*k* ^1^ [s^−1^]	*k* ^2^ [s^−1^]	*t*_½_^1^ [h]	*t*_½_^2^ [h]
**N4b**@PMMA500	Cycle 1	5.70 · 10^−4^		0.34	
**N4b**@PC	Cycle 3	1.60 · 10^−4^	2.70 × 10^−5^	1.20	7.13
**N4b**@PC	Cycle 6	9.46 · 10^−5^	2.02 × 10^−5^	2.03	9.55
**N4b**@PS	Cycle 2	2.88 · 10^−4^	3.31 × 10^−5^	0.67	5.81
**N4b**@PS	Cycle 7	5.73 · 10^−5^	1.01 × 10^−5^	3.36	19.1
**N4b**@PS	Cycle 10	4.15 · 10^−5^	5.70 × 10^−6^	4.64	33.8

Given that PS was the optimal host for **N4b** and best preserved the properties of the photoswitch, longer cycling studies were necessary to ascertain the effects on NBD@PS, and most importantly on the kinetics for the back‐reaction. To quantify the effect on the rate of conversion over successive cycles we selected **N4c**, which has a faster rate of back‐conversion and therefore should allow for faster cycling. This investigation was also undertaken using different loadings of **N4c** (0.005–0.5 wt%) into PS to probe whether the concentration of the photoswitch also played a role. Composite **N4c**@PS was more colored than **N4b**@PS on the account of a greater degree of polarization in the π‐system and had a higher extinction coefficient. Compound **Q4c** has a shorter half‐life, which made it difficult to obtain a spectrum of fully converted **Q4c**@PS.

**Figure 4:**
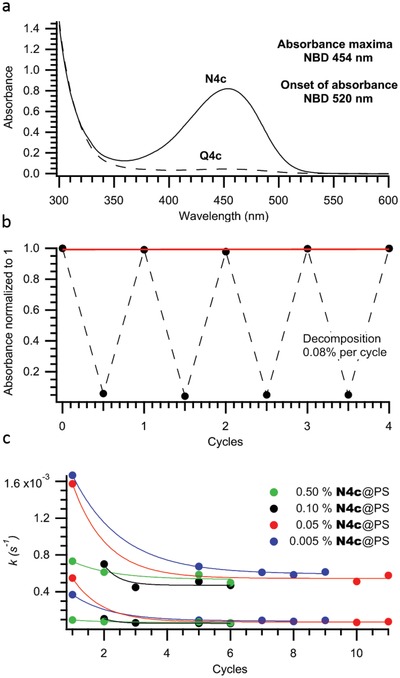
a) 0.1 wt% **N4c**@PS before and after irradiation. b) Performance of **N4c**@PS with a weight percentage of 0.5% **N4c** subjected to photothermal cycling. c) Kinetics for 0.005–0.50 wt% **Q4c→N4c**@PS for multiple cycles showing the two reaction constants for the double exponential fit.

Photoswitch **N4c** was loaded into PS at four different concentrations, and the cyclability showed a decomposition per cycle of 0.02– 0.45% for the system (**Figure** 4b); however, this was not proportionate to the amount of **N4c** introduced to PS. Unlike for **N4b**@PS, it was not possible to obtain full conversion for the more concentrated samples, as the rate of back‐conversion is too fast. More surprising was the change in the conversion rate going from **Q4c**@PS→**N4c**@PS. As seen with **N4b**@PS, the back‐conversion rate slowed down over several cycles, and gradually reached a plateau (Figure 4c) and was independent of the relative concentration of **N4c**. This could be a result of progressive structural rearrangement of the NBD within the material as **Q4c** should occupy less space than **N4c**. After several cycles there are still two rate constants, which average out at *t*_½_ 1:22 min and *t*_½_ 2:178 min for the half‐lives for **Q4c**@PS at all measured concentrations. When examining the films with an optical microscope, it was found that small crystallites had formed within the composite films, suggestive of photoswitches aggregation, which could be related to the change in rate of back‐conversion. It should be noted that the measurements were performed at ambient temperature, well below the glass transition (*T*_g_) of polystyrene (*T*_g_ (PS) ≈ 100 °C).

In order to demonstrate that these composites can not only absorb sunlight but also release the energy as heat, four different QC@PS films were prepared with a higher loading of the active molecule. Four NBDs were selected consisting of **N4b–d** and the symmetric dimer **NN4e**. Each sample was firstly irradiated under a solar simulator and immediately measured by differential scanning calorimetry (DSC). The polymer composite was recovered from the DSC pan to investigate the cyclability, then placed in a new DSC pan and subjected to two further irradiation‐heat release cycles. The heat release was measured using a gradient of 2 °C min^−1^ and the results are summarized in **Table** 3. The DSC analysis method was conducted at a markedly slower heating rate than what is normally used for measuring of heat release of neat QCs so as to avoid heating the sample above the temperatures where phase changes of the material could interfere with the anticipated exotherm. For **N4b**@PS, it was found that the heat release performance of corresponding QC remained unaffected after three cycles, when each DSC cycle heated the material to 90 °C, which is just below the glass transition temperature of the polystyrene matrix. Not surprisingly, it was not possible to obtain and analyze **Q4c**@PS, as the material back‐converted too rapidly. Material **Q4d**@PS was also measured in the DSC, though this sample could not be cycled as **N4b**@PS, as heating of the compound to 90 °C did not result in a full back‐conversion of **N4b**. Upon heating to 110 °C, we observed a full release in the DSC; however, the cyclability of the switching process diminished significantly. This could be related to softening of the polymer matrix at temperatures higher than the *T*_g_, which facilitates phase separation and the formation of NBD aggregates within the polymer matrix. Composite **QQ4e**@PS was also prepared, and the heat release of the material could be measured in a similar fashion to that of **Q4d**@PS. Collectively, these materials exhibited energy densities between 30 and 50 kJ kg^−1^ (see Table 3), though this was dependent on the amount of NBD in PS. For the sake of direct comparison, molar enthalpies for the heat release are also summarized. In all examples, the energy release per mole of QC was similar. Molecule **QQ4e**, possessing two photochromic units, leads to a higher energy density (0.48 MJ kg^−1^). We note that it was not possible to measure the heat release of the neat QCs due to their fast back‐conversion in their neat forms; however, the molar heat release could be calculated from the polymer samples. These values were found to be similar to those found for cyano analogs **Q5b**,**d**, and **QQ5e** (Table 3).

**Table 3:** Heat release for QCs in PS

	Loading of NBD [wt%]	Δ*H*_storage_ of composite [kJ kg^−1^]	Δ*H*_storage_ for NBD [MJ kg^−1^]	Δ*H*_storage_ for NBD [kJ mol^−1^]
**Q4b**@PS	8.44	30.2	0.36	105
**Q4d**@PS	14.5	50.4	0.35	110
**QQ4e**@PS	10.8	51.8	0.48	216
**Q5b**[qv: 5f]			0.40	88.5
**Q5d**[qv: 11h]			0.50	118
**QQ5e**[qv: 11g]			0.77	238

**Figure 5:**
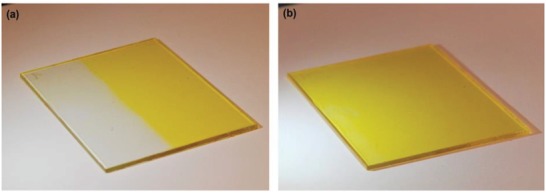
Hexamethyldisilazane treated glass (5 cm × 8 cm) coated with 70 µm (± 5 µm) thick layer of **N4b**@PS (0.8 wt% **N4b**) a) upon exposure of half of the sample to light (λ = 405 nm, ≈1700 mW, ≈1 min) and b) after back‐conversion.

To illustrate the potential of NBD–polystyrene composites for the lamination on windows, **N4b**@PS (0.8 wt% **N4b**) were cast onto glass substrates (see **Figure** 5) resulting in a film thickness of 70 µm (±5 µm). To prevent delamination of the **N4b**@PS coating we used either glass substrates which were subjected to a surface treatment with hexamethyldisilazane, or RadiSurf substrates, which comprise of a PS adhesion layer. These films converted within seconds upon exposure to sunlight, illustrating how the windows would work in real life (see Video SI in the Supporting Information).

## 3 Conclusions

In this work, new NBDs furnished with the powerful electron‐withdrawing trifluoroacetyl acceptor have been synthesized, having redshifted onset of absorbance compared to analogs with the cyano group acceptor but at the cost of shorter QC lifetimes. As a result, these new derivatives are more suited for a day to day cycling purposes, as well as having a good overlap with the solar spectrum. Nevertheless, the effect of shorter lifetimes could be mitigated by the introduction of aryl donors with steric effects in the *ortho* position, such as the 1‐naphthyl substituent, leading to systems with very long lifetimes (*t*_1/2_ = 10 months). Moreover, double systems bearing two NBD units attached to a central phenylene bridge lead to a good quantum yield and unlike former observations of similar compounds,[qv: 11g] the second photoconversion isomerization did not experience lowered quantum yields. Further, the combination of improved solar spectrum match, quantum yield, and energy storage leads to an estimated solar energy storage efficiency of up to 3.8%.

Amount the new synthesized NBDs, photochemical properties ideally matched to a daily charge/discharge cycle compounds were studied in polymer films, and it was found that polystyrene provided an attractive host, where the photoswitches could be subjected to multiple cycling without experiencing significant degradation. Molar energy storage enthalpies for the photoswitch‐PS composite were comparable to the neat NBDs, which have energy storage enthalpies in the range of 105–198 kJ mol^−1^ per NBD unit, corresponding to energy densities in the 0.34–0.48 MJ kg^−1^ range for the neat NBDs and 2.7–3.8 kJ kg^−1^ in the 0.8 wt% composites.

This study has demonstrated that NBD dispersed in a polystyrene matrix can absorb sunlight, and over time release the energy as heat. This is the first step in making window tinting that contains NBD MOST systems, and shows great promise for implementation of such a technology with the aim of energy saving.

## 4 Experimental Section

*General Experimental*: Cyclopentadiene was distilled by cracking dicyclopentadiene over iron filings and storing at −80 °C prior to use. Tetrahydrofuran used was distilled over a sodium/benzophenone couple. All other chemicals were used as purchased from commercial sources. Cold baths at −84 and −41 °C were made from liquid nitrogen/ethyl acetate and liquid nitrogen/acetonitrile slurries respectively, while −78 °C was made from dry ice and acetone. Purification of products was carried out by flash chromatography on silica gel (40−63 µm, 60 Å). Thin layer chromatography (TLC) was carried out using aluminum sheets precoated with silica gel. Infrared (IR) spectra recorded on a Perkin‐Elmer Frontier FT‐IR instrument as films evaporated from CDCl_3_ onto an ATR attachment. ^1^H NMR (400 MHz) and ^13^C NMR (100 MHz) spectra were recorded on a Varian 400 MHz instrument, or ^1^H NMR (500 MHz) and ^13^C NMR (125 MHz) spectra were recorded on a Varian 500 MHz instrument, or ^1^H NMR (800 MHz) spectra were recorded on a Bruker 500 MHz instrument, using the residual solvent as the internal standard (CDCl_3_, ^1^H = 7.26 ppm and ^13^C = 77.16 ppm, or toluene‐*d*_8,_^1^H = 2.090 ppm and ^13^C = 20.400 ppm). All NMR experiments were acquired at 298 K unless specified. All chemical shifts are quoted on the δ scale (ppm), and all coupling constants (*J*) are expressed in Hz. All solution based spectroscopic measurements were performed in a 1 cm path length cuvette on either a Cary 60 Bio or a Cary 100 UV–vis spectrophotometer, scanning the wavelength from 600 to 290 nm coupled with Peltier temperature control. Photoswitching experiments were performed using a Vilber Lourmet TLC lamp with a wavelength at 365 nm at 610 µw cm^−3^ or Thorlabs LED M365F1. Photoswitching for wavelengths 340, 405, and 455 nm were performed using Thorlabs LED M340L4, M405L3, and M455L3, respectively. The thermal back‐reaction was performed by heating the sample (cuvette) by a Peltier unit in the UV–vis spectrophotometer. Quantum Yields were measured by the published procedure using a high concentration regime (absorbance above 2 at wavelength of irradiation) using potassium ferrioxalate and tris‐phenanthroline iron(II) complex as a chemical actinometer.^17^

Polystyrene (*M*_W_ = 192 000 g mol^−1^), poly(Bisphenol A carbonate) (*M*_W_ ≈ 45 000 g mol^−1^), and polyvinylidenechloride (GoodFellow, powder, mean particle size <180 µm) were purchased from Sigma Aldrich. The molecular weight of polyvinylidenechloride (*M*_W_ = 52 700 g mol^−1^) was measured in 1,2,4‐trichlorobenzene with Snatox stabilizer at 150 °C using an Agilent PL‐GPC 220 Integrated HT‐GPC with a refractive index detector and online viscometer using three Plgel 10 µm MIXED‐B LS 300 × 7.5 mm columns with a universal calibration against polystyrene standards. Polymethylmethacrylate (*M*_W_ = 100 000 g mol^−1^) was purchased from Polysciences Inc. NBD–polymer composites were fabricated by mixing of the respective NBD into a solution of the respective polymer in dichloromethane (*c* = 100 g L^−1^). The mixtures were poured into petri dishes and were allowed to dry at 25 °C overnight. All polymer based spectroscopic measurements were performed by attaching the polymer samples to a sample holder, ensuring the measurements were made at the exact same spot of the polymer sample every time, using a Cary 60 Bio UV–vis spectrophotometer, scanning the wavelength from 600 to 300 nm. All melting points and heat release of polymer samples were recorded with a Mettler Toledo DSC 2 apparatus, with a heating rate of 2 °C min^−1^. HRMS spectra by either atmospheric pressure chemical ionization (APCI) or electrospray ionization (ESI) were acquired using an Agilent 1260 Infinity instrument fitted with an Agilent 6120 quadrupole. Elemental analyses were performed at London Metropolitan University. Terminal alkyne precursors to **3a**,**c–e**,^18^ **3b** 16 were made by their respective literature methods.

*4‐(4‐(N,N‐Dimethylamino)phenyl)‐1,1,1‐trifluorobut‐3‐yn‐2‐one (3c)*: To a stirring solution of 4‐ethynyl‐*N*,*N*‐dimethylaniline (991 mg, 6.83 mmol) in dry THF (50 mL) at −84 °C was added dropwise *n*‐BuLi (3.0 mL, 2.5 m in hexanes, 7.5 mmol) and the reaction was allowed to stir 15 min, before allowing the temperature to be raised to 0 °C for 30 min. The reaction mixture was cooled back down to −84 °C, and ethyl 2,2,2‐trifluoroacetate (1.6 mL, 13.5 mmol) was added. The cold bath was removed and the reaction contents were allowed to warm to ambient temperature during the course of 16 h. Then the reaction was quenched with saturated NH_4_Cl (60 mL) and the phases was separated. The aqueous phase was extracted with CH_2_Cl_2_ (2 × 50 mL) and the combined organic phases dried over Na_2_SO_4_, filtered and the solvent was removed under reduced pressure. The residue was purified by a short column (25% CH_2_Cl_2_/petroleum sprit) to give **3c** (1.23 g, 75%) as a yellow solid. *R*_f_ = 0.31 (60% CH_2_Cl_2_/hexane). M.p. = 103.5–104.2 °C. IR: υ_max_ = 2947, 2920, 2183, 2159, 1690, 1599, 1533 cm^−1^. ^1^H NMR (500 MHz, CDCl_3_): δ = 7.53 (d, *J* = 9.1 Hz, 2H), 6.64 (d, *J* = 9.1 Hz, 2H), 3.08 (s, 6H) ppm. ^13^C NMR (125 MHz, CDCl_3_): δ = 166.62 (q, *J* = 41.0 Hz), 152.99, 136.56, 115.42 (q, *J* = 288.7 Hz), 111.72, 107.30 (q, *J* = 1.1 Hz), 103.13, 86.53, 40.09 ppm. HRMS (ESI, +ve) calcd for C_12_H_10_F_3_NO [(M+H)^+^]: *m*/*z* = 242.0787; exp 242.0794. Analysis calcd (%) for C_12_H_10_F_3_NO (241.21): C 59.75, H 4.18, N 5.81; found: C 59.99, H 4.17, N 5.79.

*1,1,1‐Trifluoro‐4‐(naphthalen‐1‐yl)but‐3‐yn‐2‐one (3d)*: To a solution of 1‐ethynylnaphthalene (1.09 g, 7.16 mmol) in dry THF (50 mL) at −84 °C under argon was added *n*‐BuLi (3.15 mL, 2.5 m in hexane, 7.87 mmol) and the reaction was allowed to stir 20 min, before being stirred at 0 °C for 1 h. The contents of the vessel were cooled to −84 °C and ethyl 2,2,2‐trifluoroacetate (1.7 mL, 14.3 mmol) was added. The reaction mixture was allowed to slowly reach ambient temperature overnight, after which time saturated aqueous NH_4_Cl (60 mL) and H_2_O (50 mL) were added. The phases were separated and the aqueous phase was extracted with CH_2_Cl_2_ (2 × 50 mL). The combined organic phases were dried over Na_2_SO_4_, filtered and the solvent was removed by rotary evaporation. The residue was purified by a short silica gel column (20% CH_2_Cl_2_/petroleum sprit) to afford **3d** (1.60 g, 90%) as yellow solid. *R*_f_ = 0.21 (20% CH_2_Cl_2_/*n*‐heptane). M.p. = 57.9–58.7 °C. IR = 3062, 2182sh, 2168, 1694, 1586, 1576, 1507 cm^−1^. ^1^H NMR (500 MHz, CDCl_3_): δ = 8.28 (ddd, *J* = 8.4, 1.3, 0.6 Hz, 1H), 8.07 (dd, *J* = 8.3, 1.2 Hz, 1H), 7.97 (dd, *J* = 7.2, 1.2 Hz, 1H), 7.93 (ddd, *J* = 8.2, 1.3, 0.6 Hz, 1H), 7.69 (ddd, *J* = 8.4, 6.9, 1.3 Hz, 1H), 7.61 (ddd, *J* = 8.2, 6.9, 1.3 Hz, 1H), 7.53 (dd, *J* = 8.3, 7.2 Hz, 1H) ppm. ^13^C NMR (125 MHz, CDCl_3_): δ = 167.26 (q, *J* = 42.1 Hz), 135.32, 133.95, 133.76, 133.14, 128.95, 128.66, 127.52, 125.39, 125.34, 115.68, 115.36 (q, J = 288.4 Hz), 99.55 (q, *J* = 1.0 Hz), 88.33 ppm. HRMS (APCI, +ve) calcd for C_14_H_7_F_3_O [(M+H)^+^]: *m*/*z* = 249.0522; exp 249.0527. Analysis calcd (%) for C_14_H_7_F_3_O (248.20): C 67.75, H 2.84; found: C 67.62, H 2.64.

*2‐Trifluoroacetyl‐3‐(4‐methoxyphenyl)norbornadiene (N4b)*: A vial suitable for microwave reactions was charged with **3b** (506 mg, 2.22 mmol), cyclopentadiene (1.0 mL, 12 mmol), BHT and chlorobenzene (1.5 mL) and was heated for 1 h at 100 °C. The reaction was followed by TLC analysis (50% CH_2_Cl_2_/*n*‐hexane) indicating an incomplete reaction and cyclopentadiene (0.5 mL, 6.05 mmol) was added to the vial and the reaction mixture was heated for a further 20 h at 100 °C. The product was purified by flash column chromatography (gradient elution of *n*‐hexane to 45% CH_2_Cl_2_/*n*‐hexane) to give **N4b** (337 mg, 57%) as a yellow solid. *R*_f_ = 0.35 (50% CH_2_Cl_2_/*n*‐hexane). M.p. = 40.3–41.3 °C. IR: υ_max_ = 3073, 3002, 2974sh, 2945, 2874, 2841, 1679, 1602, 1573, 1541, 1504 cm^−1^. ^1^H NMR (500 MHz, CDCl_3_): δ = 7.76 (d, *J* = 9.0 Hz, 2H), 6.96 (br dd, *J* = 4.9, 3.0 Hz, 1H), 6.94 (d, *J* = 9.0 Hz, 2H), 6.89 (br dd, *J* = 4.9, 3.2 Hz, 1H), 4.19 (ddtd, *J* = 3.0, 2.4, 1.6, 0.8 Hz, 1H), 3.97 (ddtd, *J* = 3.2, 2.4, 1.6, 0.7 Hz, 1H) 3.86 (s, 3H), 2.24 (dt, *J* = 7.1, 1.6 Hz, 1H), 2.17 (dt, *J* = 7.1, 1.6 Hz, 1H) ppm. ^13^C NMR (125 MHz, CDCl_3_): δ = 178.26, 176.78 (q, *J* = 34.2 Hz), 161.85, 144.02, 139.74, 137.51, 130.80, 127.51, 117.08 (q, *J* = 292.4 Hz), 113.50, 69.13, 58.94, 55.56, 51.90 (q, *J* = 3.0 Hz) ppm. HRMS (ESI, +ve) calcd for C_16_H_13_F_3_O_2_ [(M+H)^+^]: *m*/*z* = 295.0940; exp 295.0940. Analysis calcd (%) for C_16_H_13_F_3_O_2_ (294.27): C 65.31, H 4.45; found: C 65.23, H 4.58.

*2‐Trifluoroacetyl‐3‐(4‐N,N‐dimethylaminophenyl)norbornadiene (N4c)*: A solution of **3c** (309 mg, 1.28 mmol), cyclopentadiene (1 mL, 12 mmol), chlorobenzene (1.5 mL), and some BHT was sealed in a microwave tube and heated to 100 °C for 16 h. After which time, the cooled mixture was subjected to flash column chromatography (gradient elution of *n*‐hexane to 60% CH_2_Cl_2_/*n*‐hexane) to give recovered **3c** (33 mg, 11%) and **N4c** (301 mg 76%) as a red solid. *R*_f_ = 0.31 (60% CH_2_Cl_2_/*n*‐hexane). M.p. = 150.6 −151.2 °C. IR: υ_max_ = 3078, 3030, 2977, 2938, 2867, 1646, 1605, 1566, 1539 cm^−1^. ^1^H NMR (500 MHz, CDCl_3_): δ = 7.90 (d, *J* = 9.2 Hz, 2H), 6.92 (br dd, *J* = 5.0, 2.9 Hz, 1H), 6.80 (dd, *J* = 5.0, 3.2 Hz, 1H), 6.69 (d, *J* = 9.2 Hz, 2H), 4.17 (ddt, *J* = 3.2, 2.9, 1.6, 1H), 4.04 (ddtd, *J* = 3.2, 2.4, 1.6, 0.7 Hz, 1H), 3.06 (s, 6H), 2.17 (dt, *J* = 7.1, 1.6 Hz, 2H), 2.12 (dt, *J* = 7.1, 1.6 Hz, 1H ppm. ^13^C NMR (125 MHz, CDCl_3_): δ = 179.38, 175.53 (q, *J* = 33.3 Hz), 152.50, 144.06, 138.98, 133.59, 131.56, 122.22, 117.60 (q, *J* = 292.5 Hz), 110.73, 67.68, 58.17, 51.52 (q, *J* = 3.2 Hz), 40.1 ppm. HRMS (ESI, +ve) calcd for C_17_H_16_F_3_NO [(M)^·+^]: *m*/*z* = 308.1257; exp 308.1271. Analysis calcd (%) for C_17_H_16_F_3_NO (307.32): C 66.44, H 5.25, N 4.56; found: C 66.35, H 5.13, N 4.67.

*2‐Trifluoroacetyl‐3‐(1‐naphthyl)norbornadiene (N4d)*: A vessel suitable for microwave reactions was charged with **3d** (342 mg, 1.38 mmol), cyclopentadiene (1.0 mL, 12 mmol), chlorobenzene (1.5 mL) and BHT and the sealed tube heated to 100 °C for 1 h. The mixture was allowed to cool to ambient temperature and purified by flash column chromatography (gradient elution of *n*‐hexane to 30% CH_2_Cl_2_/*n*‐hexane) followed by a second column (30% toluene/*n*‐hexane) to furnish **N4d** (388 mg, 90%) as a yellow oil. *R*_f_ = 0.31 (30% CH_2_Cl_2_/*n*‐hexane). IR: υ_max_ = 3060, 2990, 2942, 2873, 1695, 1673, 1598, 1589, 1573, 1557, 1507 cm^−1^. ^1^H NMR (500 MHz, 313 K, CDCl_3_): δ = 7.88 (dd, *J* = 22.5, 8.2 Hz, 2H), 7.58–7.40 (m, 3H), 7.40 (br s, 2H), 7.15 (t, *J* = 3.9 Hz, 1H), 6.98 (s, 1H), 4.34 (s, 1H), 3.94 (s, 1H), 2.62 (s, 1H), 2.34 (d, *J* = 7.0 Hz, 1H) ppm. ^13^C NMR (125 MHz, 315 K, CDCl_3_): δ = 177.69–177.43 (m), 144.13 (br), 141.38 (br), 134.77, 133.64, 130.35, 129.47, 128.83, 126.66, 126.35, 125.35, 125.17 (br), 116.32 (q, *J* = 291.2 Hz), 70.80 (br), 60.90, 52.19 (br) ppm (3C masked). HRMS (ESI, +ve) calcd for C_19_H_13_F_3_O [(M)^·+^]: *m*/*z* = 315.0991; exp 315.0993.

*4,4′‐(1,4‐Phenylene)bis(1,1,1‐trifluorobut‐3‐yn‐2‐one) (3e)*: To a solution of 1,4‐diethynylbenzene (751 mg, 5.95 mmol) in dry THF (100 mL) at −84 °C, under a nitrogen atmosphere was added dropwise *n*‐BuLi (5.0 mL, 2.5 m in hexane). The solution was allowed to heat to 0 °C in an ice bath, while a white precipitate formed. After 1 h the mixture was cooled back down to −84 °C and ethyl trifluoroacetate (3 mL, 25 mmol) was added and the reaction mixture was allowed to heat to ambient temperature overnight giving a homogenous solution. The reaction was quenched with saturated aqueous NH_4_Cl (100 mL) and extracted with Et_2_O (3 × 50 mL). The combined organic phases were dried over Na_2_SO_4_, filtered and volatiles removed under reduced pressure. The crude residue was purified by flash column chromatography (gradient elution of 25–50% CH_2_Cl_2_/*n*‐hexane) to furnish **3e** (922 mg, 49%) as a yellow solid. *R*_f_ = 0.19 (50% CH_2_Cl_2_/*n*‐heptane). M.p. = 57.9–58.7 °C. IR: υ_max_ = 2203, 1704, 1500 cm^−1^. ^1^H NMR (500 MHz, CDCl_3_): δ = 7.75 (s, 4H) ppm. ^13^C NMR (125 MHz, CDCl_3_): δ = 167.11 (q, *J* = 42.7 Hz), 134.03, 121.98, 114.86 (q, *J* = 288.3 Hz), 97.25 (q, *J* = 1.0 Hz), 85.25 ppm. HRMS (ESI, −ve) calcd for C_14_H_4_F_6_O_2_ [(M+OH)^·−^]: *m*/*z* = 335.0148; exp 335.0150. Analysis calcd (%) for C_14_H_4_F_6_O_2_ (318.17): C 52.85, H 1.27; found: C 52.69, H 1.17.

*Compound NN4e*: A sealed tube containing **3e** (456 mg, 1.43 mmol), cyclopentadiene (2 mL, 24 mmol), chlorobenzene (2 mL) and BHT was heated to 100 °C for 1 h. The ambient cooled solution was directly subjected to flash column chromatography (gradient elution of *n*‐hexane to 50% CH_2_Cl_2_/*n*‐hexane) to give **NN4e** (617 mg, 96%) as a yellow solid. *R*_f_ = 0.48 (50% CH_2_Cl_2_/*n*‐heptane). M.p. = 130.8–135.8 °C. IR: υ_max_ = 2989, 2946, 2875, 1688, 1573, 1552 cm^−1^. ^1^H NMR (500 MHz, CDCl_3_): δ = 7.66 (s, 4H), 7.66 (s, 4H), 7.02–6.99 (m, 4H), 6.96–6.92 (m, 4H), 4.24–4.20 (m, 4H), 3.99–3.96 (m, 4H), 2.31 (dt, *J* = 7.2, 1.6 Hz, 2H), 2.31 (dt, *J* = 7.2, 1.7 Hz, 2H), 2.23 (dt, *J* = 7.1, 1.6 Hz, 4H) ppm. ^13^C NMR (125 MHz, CDCl_3_): δ = 177.43 (q, *J* = 35.0 Hz), 177.41 (q, *J* = 34.9 Hz), 176.74, 143.88, 141.20, 141.18, 140.38, 140.36, 136.43, 136.42, 127.82, 127.81, 116.72 (q, *J* = 292.1 Hz), 70.14, 70.12, 59.07, 52.37–52.20 (m) ppm (6C masked). HRMS (ESI, +ve) calcd for C_24_H_16_F_6_O_2_ [(M+H)^+^]: *m*/*z* = 451.1127; exp 451.1132. Analysis calcd (%) for C_24_H_16_F_6_O_2_ (450.38): C 64.00, H 3.58; found: C 63.79, H 3.47.

*Coating of Glass Slide with N4b@PS*: For the coating of the **N4b**@PS composite on glass slides, **N4b** was admixed to a solution of polystyrene in toluene (*c* = 200 g L^−1^) to yield a dry composite with 0.8 wt% **N4b** in PS. The glass substrates were cleaned with acetone and isopropanol and then treated with an oxygen plasma at 50 W for 1 min. In a next step the activated glass slides were placed onto a hotplate at 70 °C, next to a recipient with 100 µL of hexamethyldisilazane and both were covered with an air tight lid. After 30 min the modified glass substrates were removed and rinsed with acetone. Alternatively, the composite was deposited onto glass slides (75 mm × 25 mm) coated with a RadiSurf PS adhesion layer provided by RadiSurf ApS, Denmark, cleaned with acetone and isopropanol. The blade coating was performed at room temperature with a RK control coater, with a blade height of 30 µm at speed setting 2.

*Single Crystal X‐Ray Crystallography*: A crystal of **N4c** was mounted under paratone‐N oil on a nylon loop, and X‐ray diffraction data were collected at 150(2) K with Mo Kα radiation (λ = 0.7107 Å) on an Oxford Diffraction X‐calibur small molecule diffractometer.^19^ The data set was corrected for absorption and the structure solved by direct methods using SHELXS‐2014 and refined by full matrix least‐squares on *F* ^2^ by SHELXL‐2014, interfaced through the program X‐Seed.^20^ All nonhydrogen atoms were refined anisotropically, and hydrogen atoms were included as invariants at geometrically estimated positions. Crystal data for **N4c**: C_17_H_16_NOF_3_, F.w. 307.31, monoclinic, *P*2_1_/*n*, *a* 8.4034(4), *b* 7.9741(4), *c* 21.4594(11) Å, β 97.015(4)°, *V* 1427.20(12) Å^3^, *Z* = 4, *D*_calc_ = 1.430 Mg m^−3^, μ 0.115 mm^−1^, *F*(000) 640, crystal size 0.58 × 0.46 × 0.14 mm^3^, θ range for data collection 3.53 to 29.28°, Ind. reflns 3459, Obs. reflns 2355, *R*_int_ 0.0478, *GoF* 1.055, *R*_1_ [I>2σ(I)] 0.0554, *wR*_2_ (all data) 0.1181, largest diff. peak and hole 0.219 and −0.248 e Å^−3^. Some disorder was apparent in the crystal structure corresponding to the two NBD isomers and this was modelled with a 0.59:0.41 site occupancy ratio. CCDC 1875800 contains the supplementary crystallographic data for this paper. These data can be obtained free of charge from The Cambridge Crystallographic Data Centre via www.ccdc.cam.ac.uk/data_request/cif.

## Conflict of Interest

The authors declare no conflict of interest.

## Supporting information

Supplementary

Supplementary
