# Supplementary material for: Solar Energy Storage by Molecular Norbornadiene–Quadricyclane Photoswitches: Polymer Film Devices
Source: Adv Sci (Weinh). 2019 Apr 25;6(12):1900367. doi: 10.1002/advs.201900367 (PMC6662068; doi:10.1002/advs.201900367)
Supplement: Supplementary file 1 — Supplementary [file ADVS-6-1900367-s002.pdf]

Copyright WILEY-VCH Verlag GmbH & Co. KGaA, 69469 Weinheim, Germany, 2019.

ADVANCED SCIENCE
Open Access

Supporting Information

for *Adv. Sci.*, DOI: 10.1002/advs.201900367

Solar Energy Storage by Molecular Norbornadiene–Quadricyclane Photoswitches: Polymer Film Devices

Anne Ugleholdt Petersen, Anna I. Hofmann, Méritxell Fillols, Mads Mansø, Martyn Jevric, Zhihang Wang, Christopher J. Sumby, Christian Müller, and Kasper Moth-Poulsen**

Copyright WILEY-VCH Verlag GmbH & Co. KGaA, 69469 Weinheim, Germany, 2019.

Supporting Information

Solar energy storage by molecular Norbornadiene-Quadricyclane photoswitches: towards polymer film devices

Anne Ugleholdt Petersen, Anna I. Hofmann, Métritxell Fillols, Mads Mansø, Martyn Jevric, Zhihang Wang, Christopher J. Sumby, Christian Müller, Kasper Moth-Poulsen

Contents

NMR Spectra	page S3
UV-Vis absorbance and kinetic study in solution	page S35
NMR studies of photo conversion of **NN4e** and **NN11**	page S44
Photoisomerisation quantum yields for **N4b-d**, **NN4e**, **NN11**	page S52
Incorporation of NBD into a polymer	page S55
UV-Vis absorbance and kinetic study of **N4b** in polymers	Page S56
UV-Vis absorbance and kinetic study of **N4c** in PS	Page S58
Heat release measurements for QCs in polymers	Page S60
Theoretical maximum energy storage	Page S67

NMR spectra

Compound **3c**

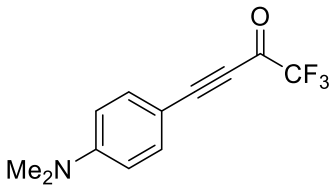

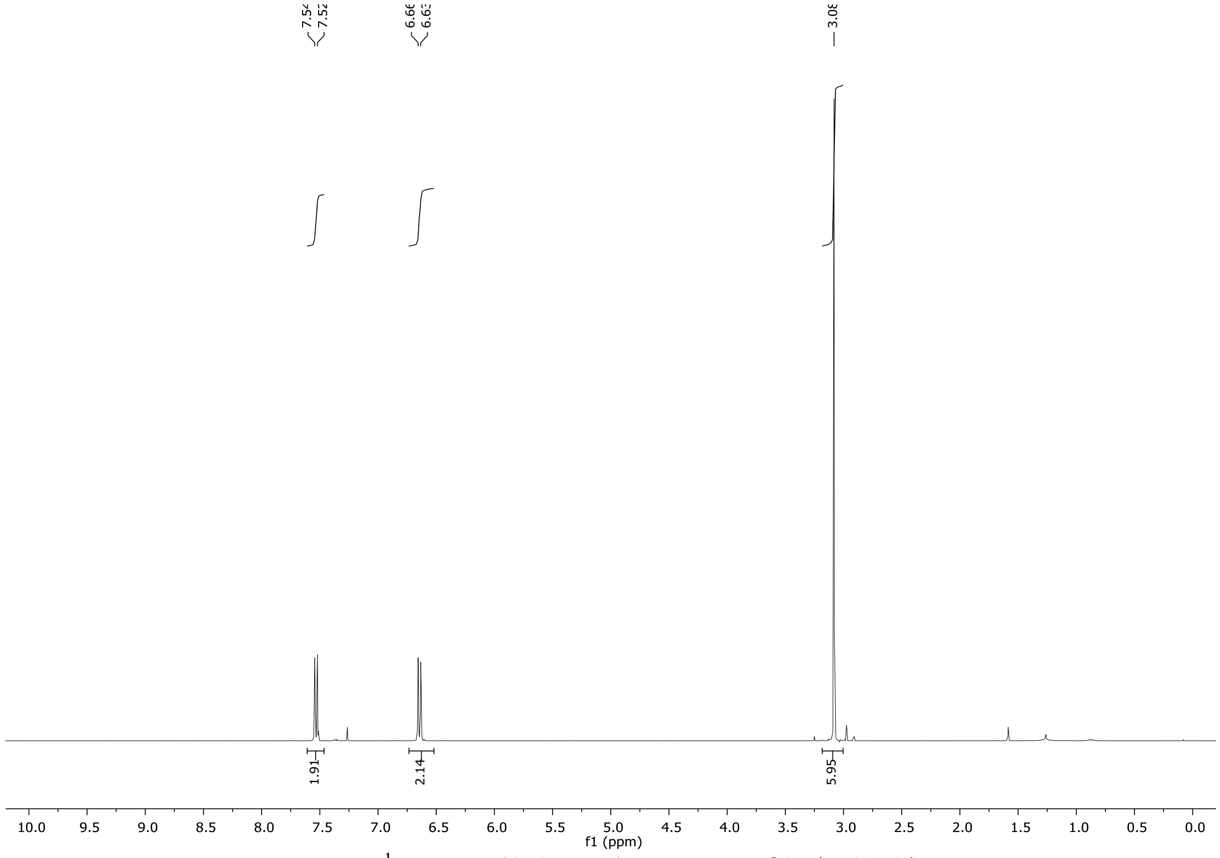

^{1}H NMR (400 MHz) spectrum of **3c** in $CDCl_3$

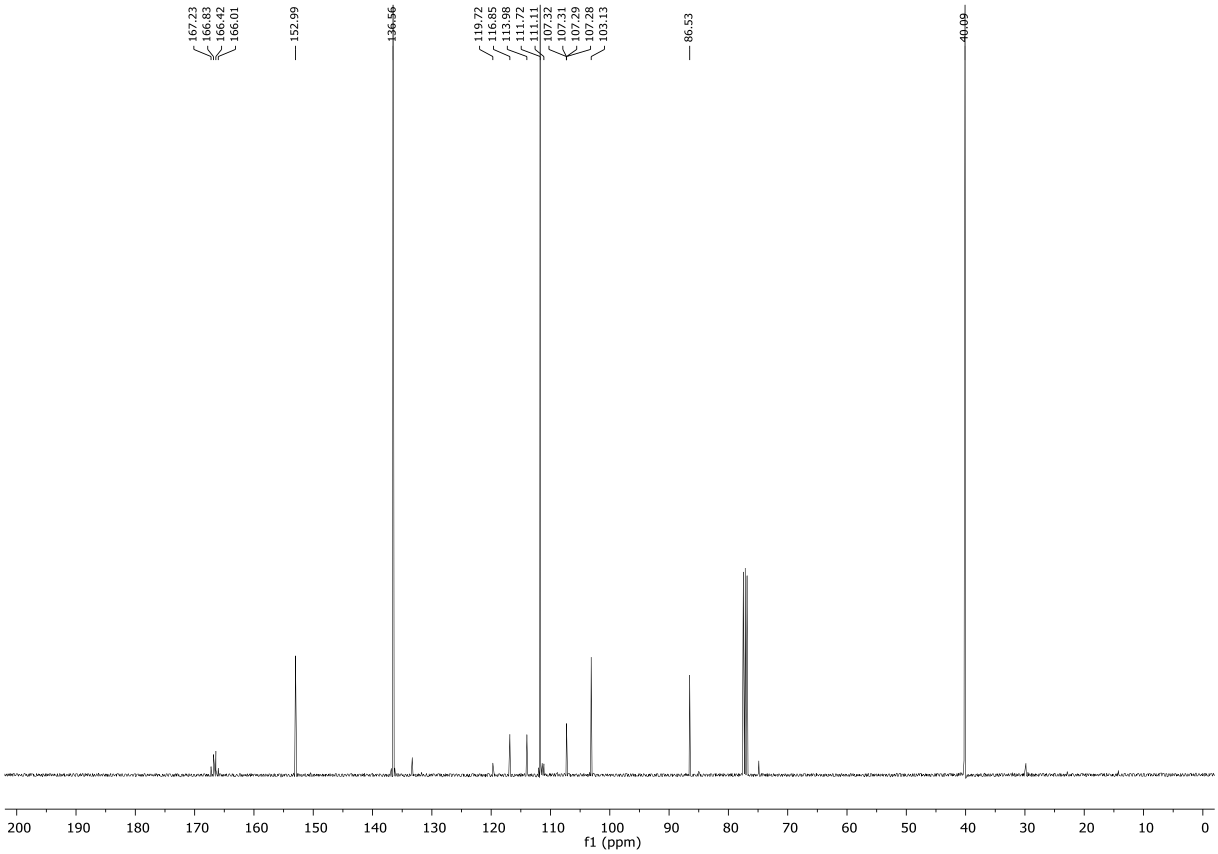

^{13}C NMR (100 MHz) spectrum of **3c** in $CDCl_3$

Compound **3d**

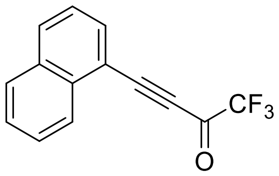

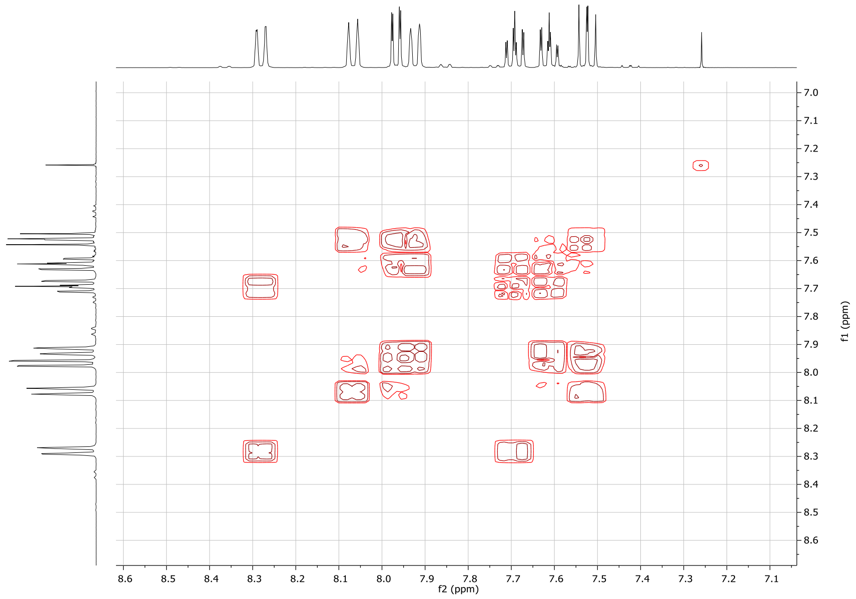

COSY NMR (400 MHz) 2D spectrum of **3d** in $CDCl_3$

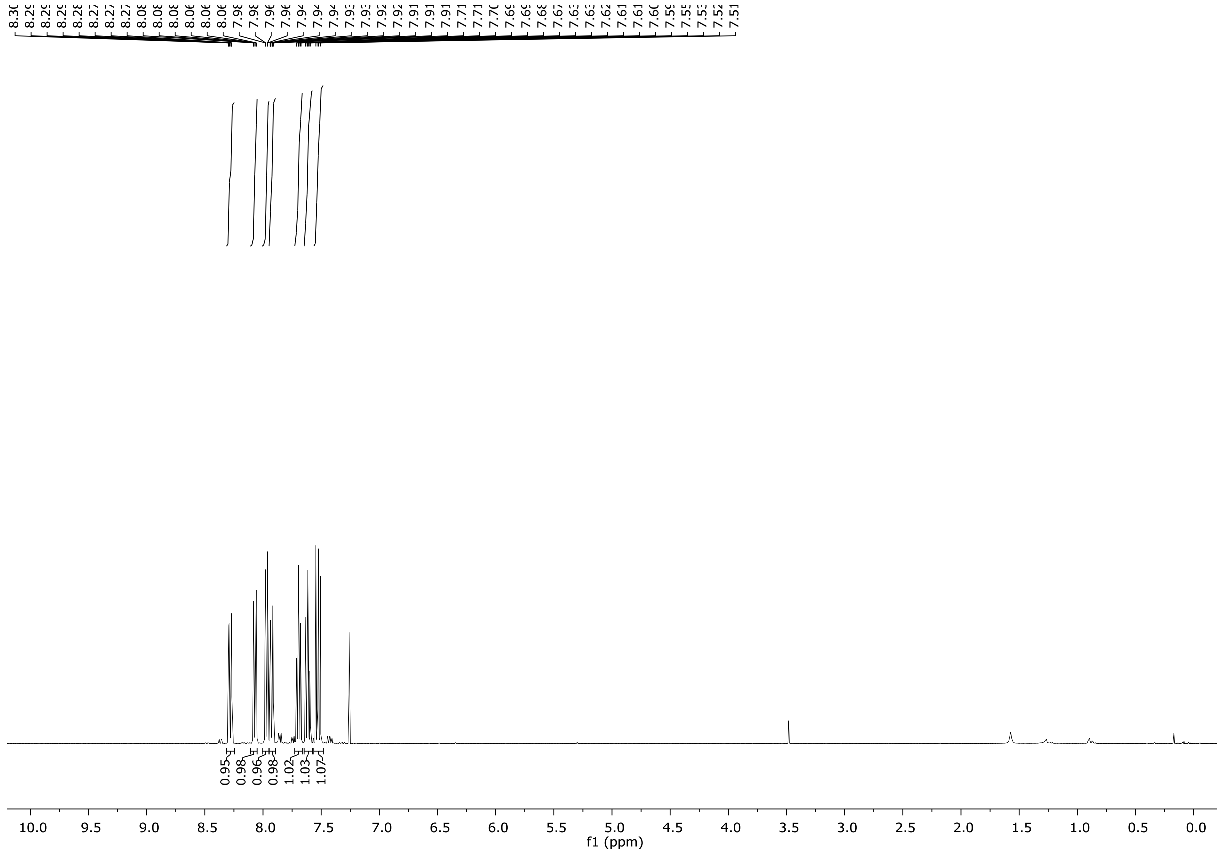

^{1}H NMR (400 MHz) spectrum of **3d** in $CDCl_3$

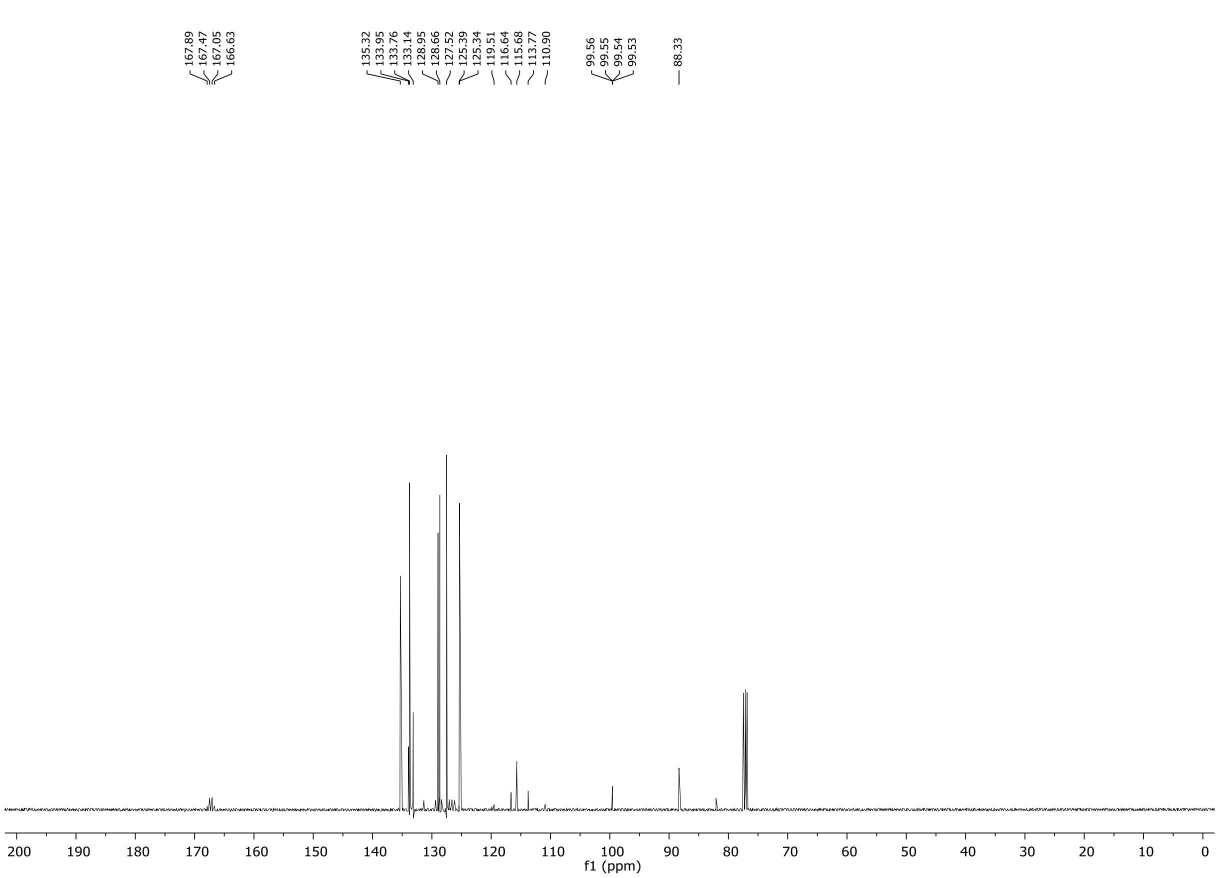

^{13}C NMR (100 MHz) spectrum of **3d** in $CDCl_3$

Compound **3e**

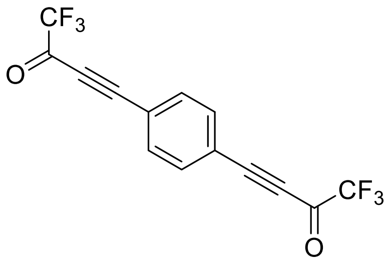

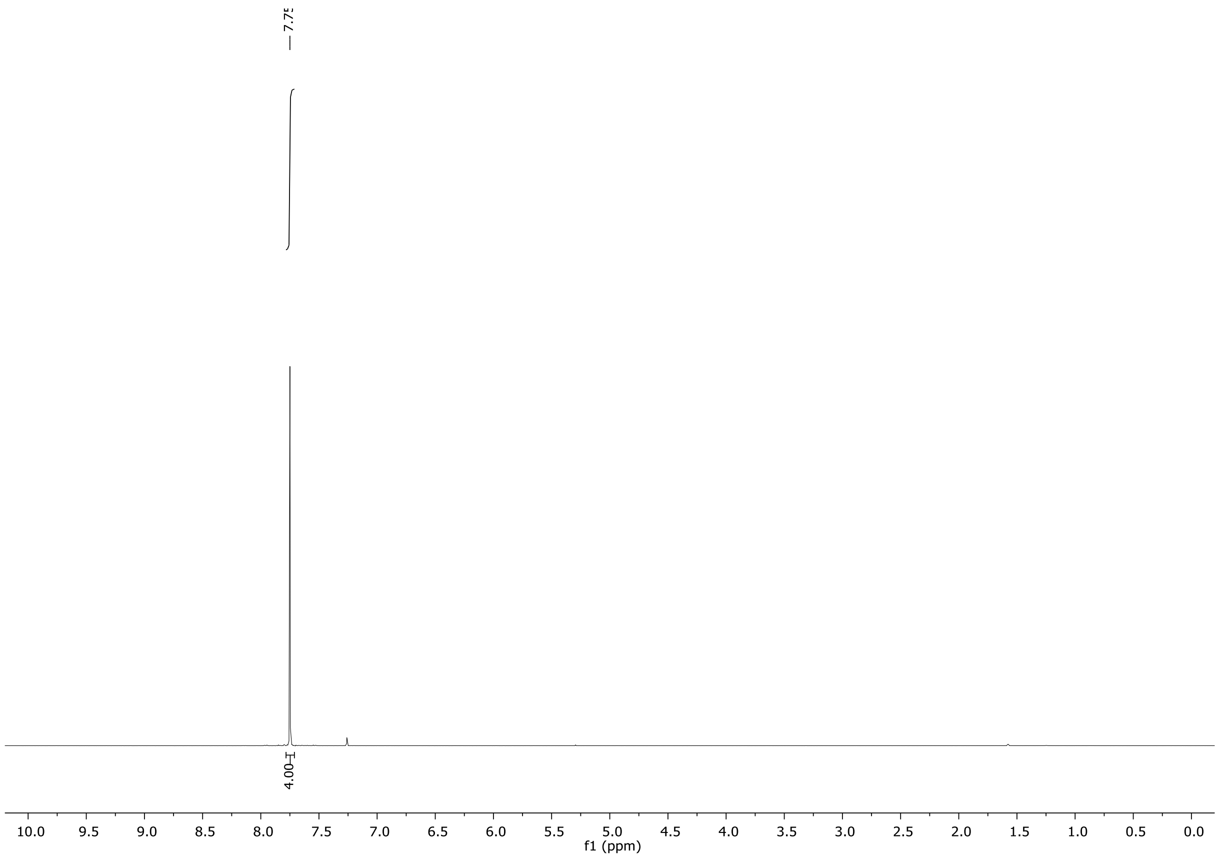

^{1}H NMR (400 MHz) spectrum of **3e** in $CDCl_3$

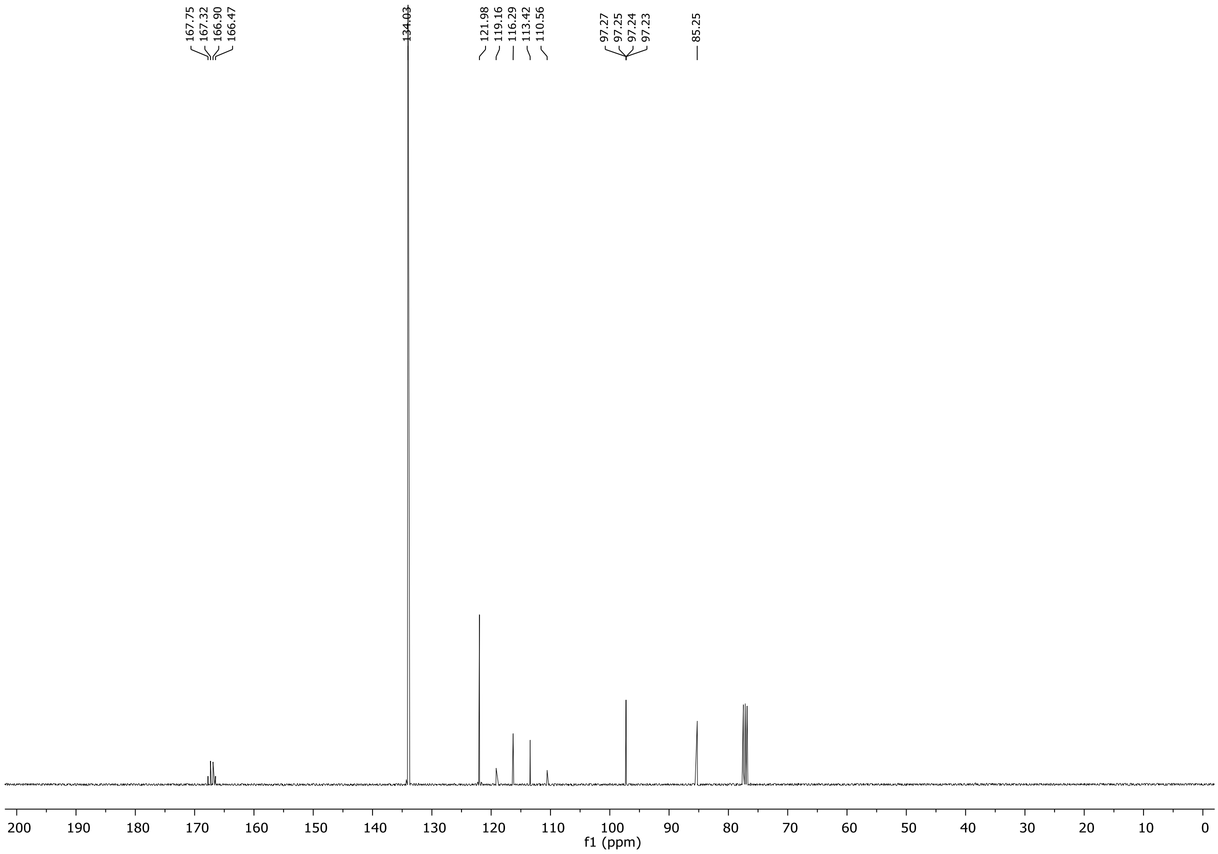

^{13}C NMR (100 MHz) spectrum of **3e** in $CDCl_3$

Compound **N4b**

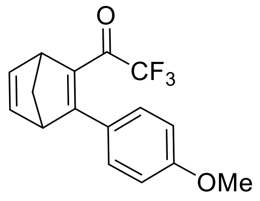

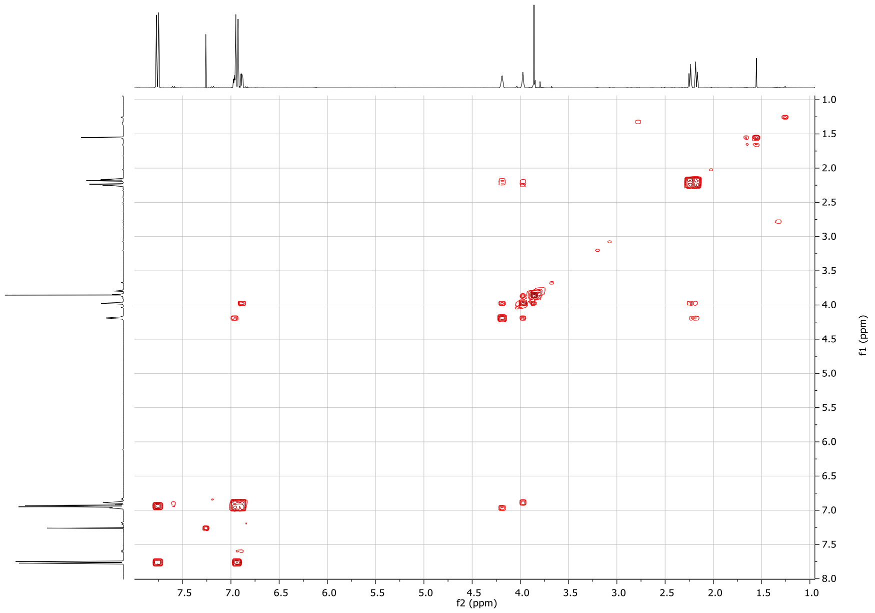

COSY NMR (400 MHz) 2D spectrum of **N4b** in $CDCl_3$

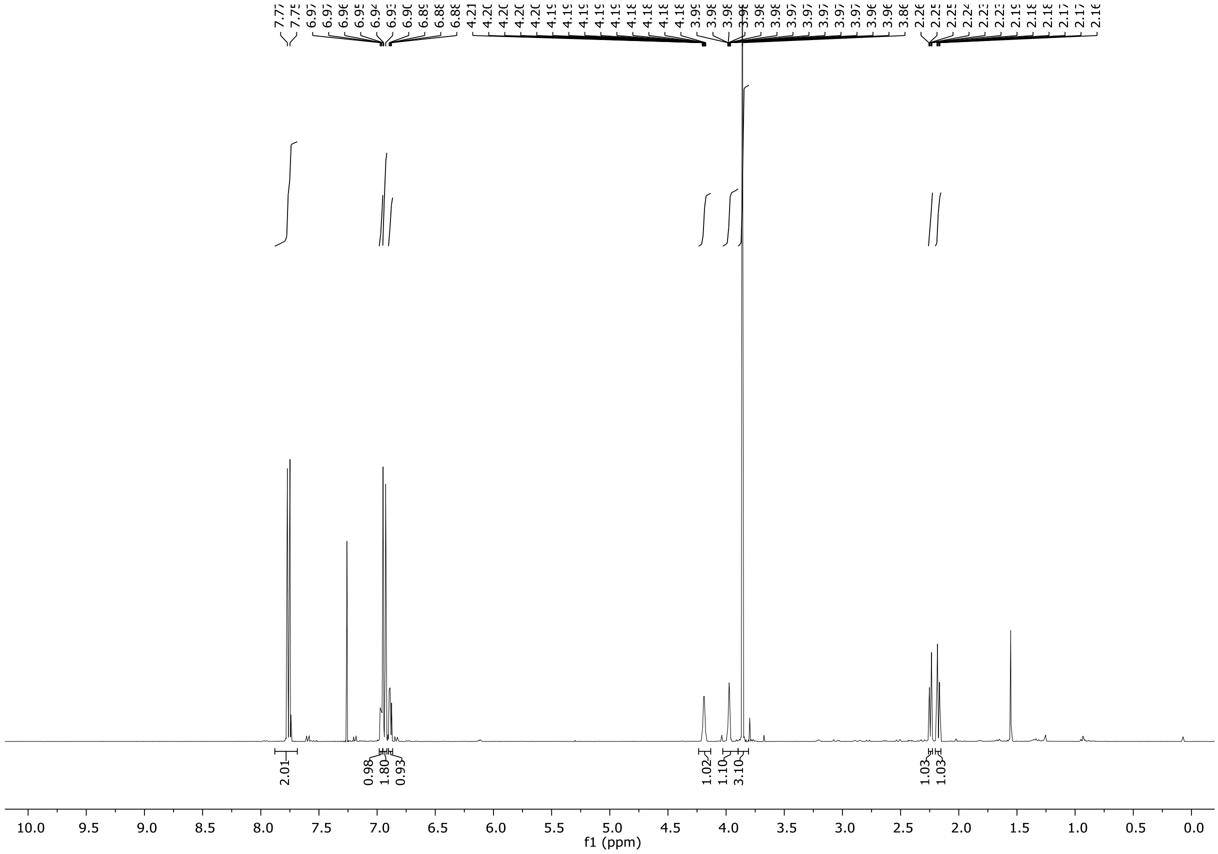

^{1}H NMR (400 MHz) spectrum of **N4b** in $CDCl_3$

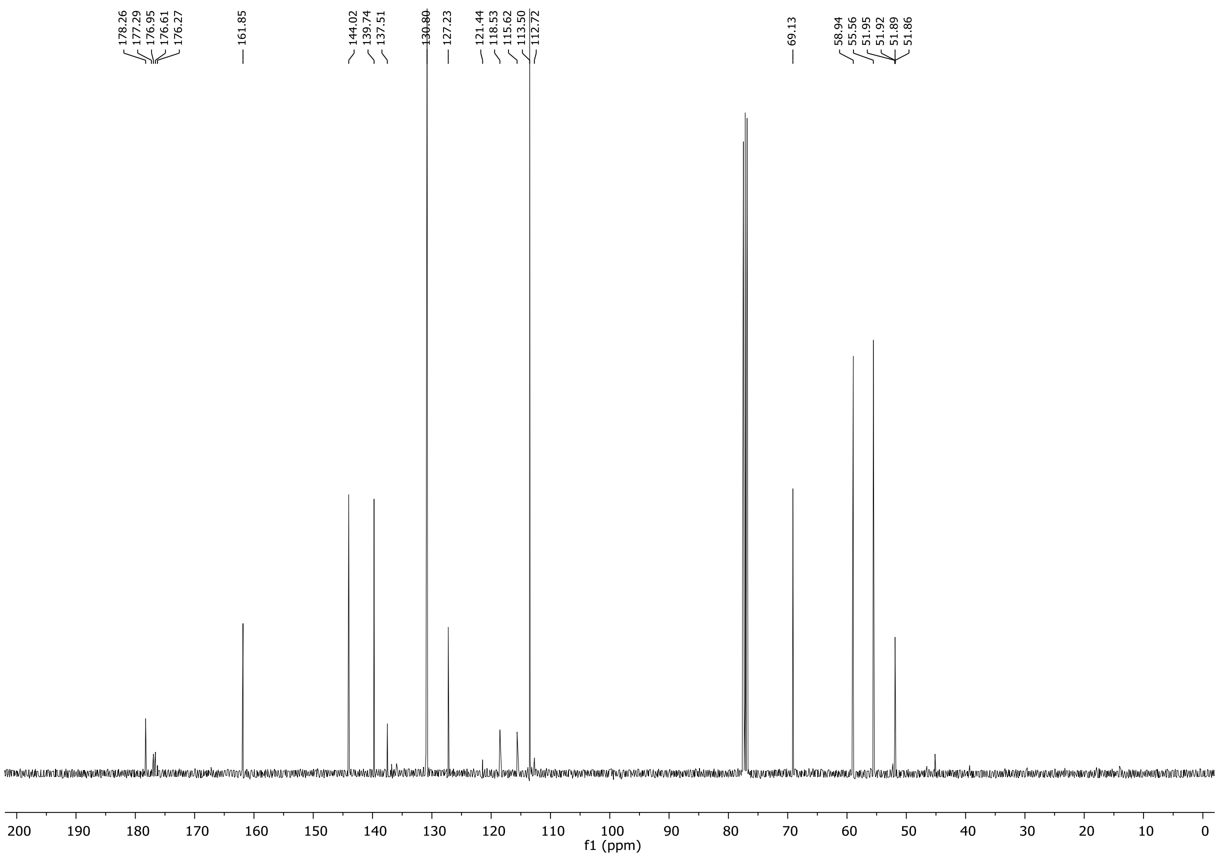

^{13}C NMR (100 MHz) spectrum of **N4b** in $CDCl_3$

Compound **N4c**

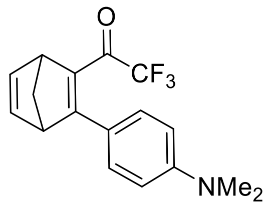

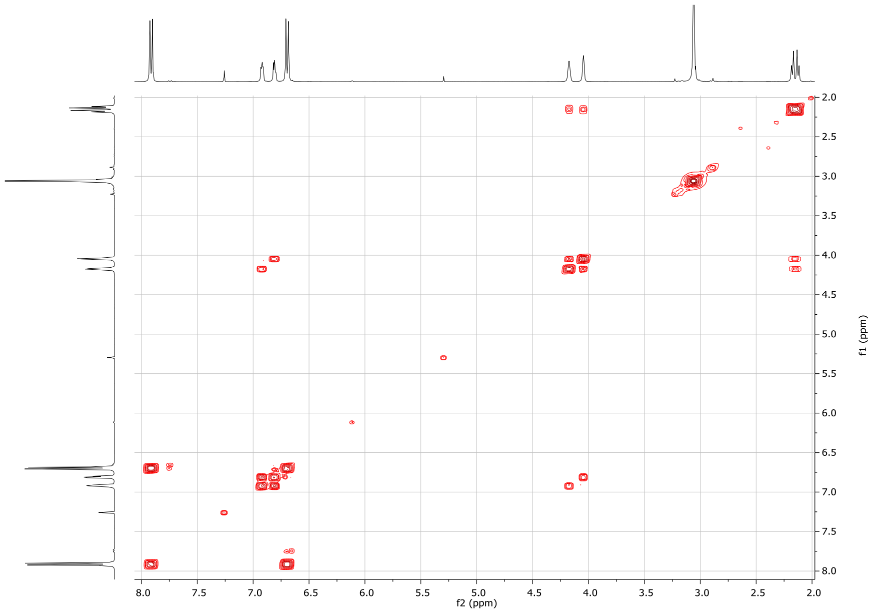

COSY NMR (400 MHz) 2D spectrum of **N4c** in $CDCl_3$

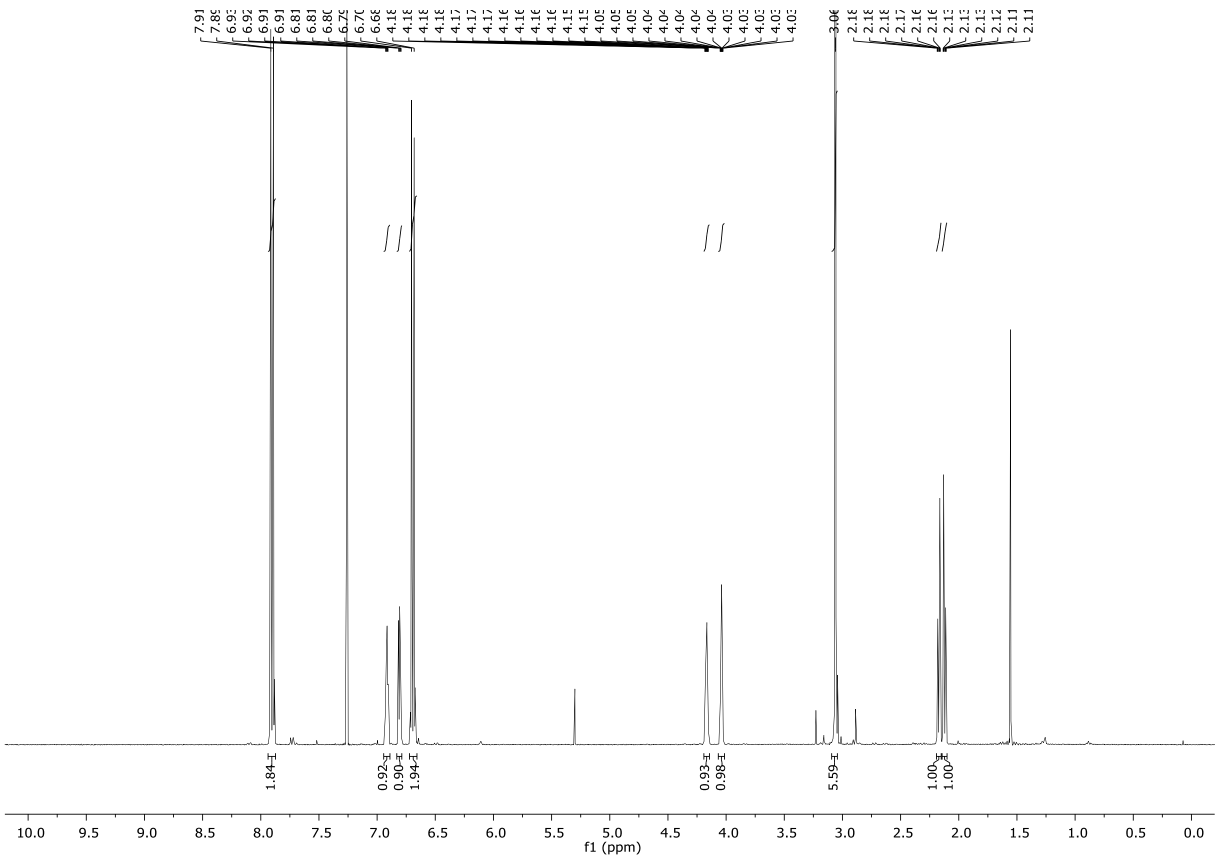

^{1}H NMR (400 MHz) spectrum of **N4c** in $CDCl_3$

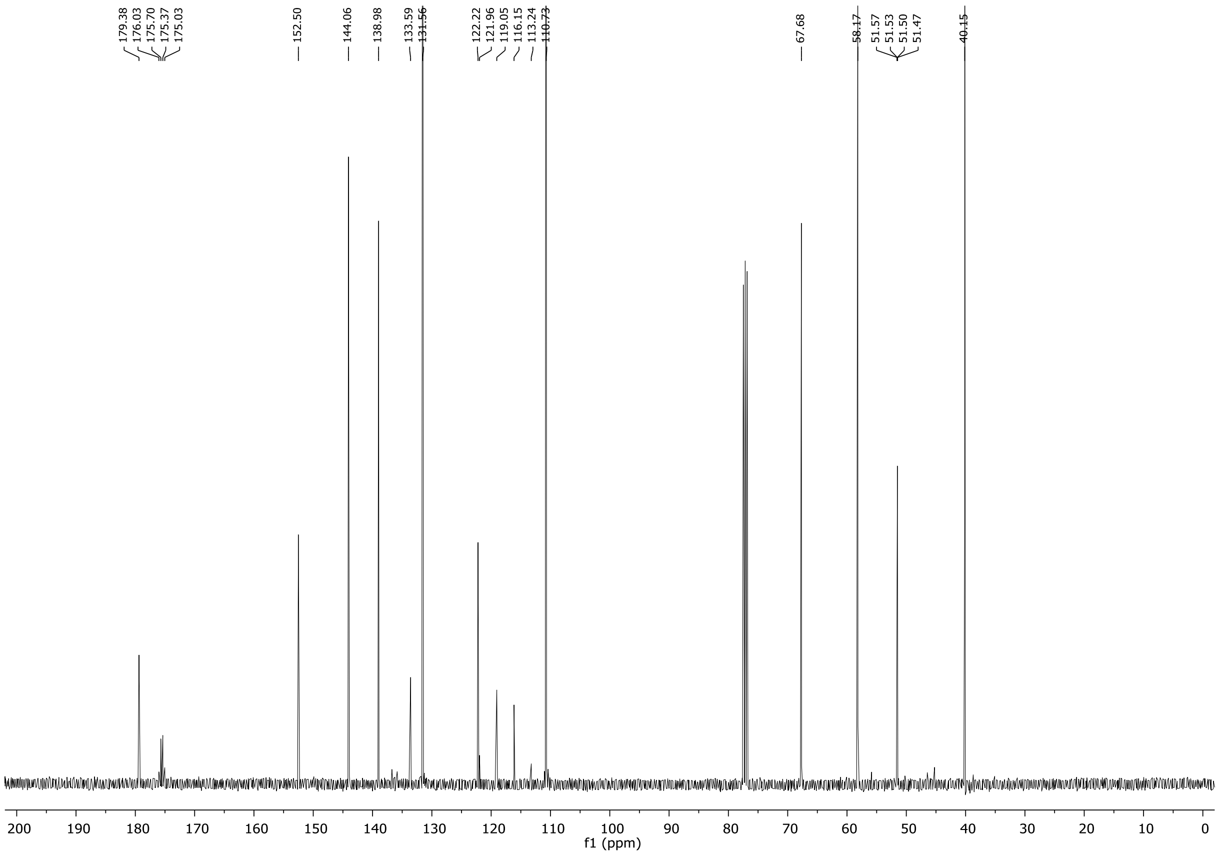

^{13}C NMR (100 MHz) spectrum of **N4c** in $CDCl_3$

Compound **N4d**

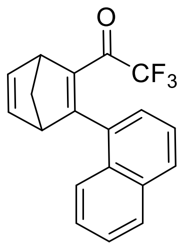

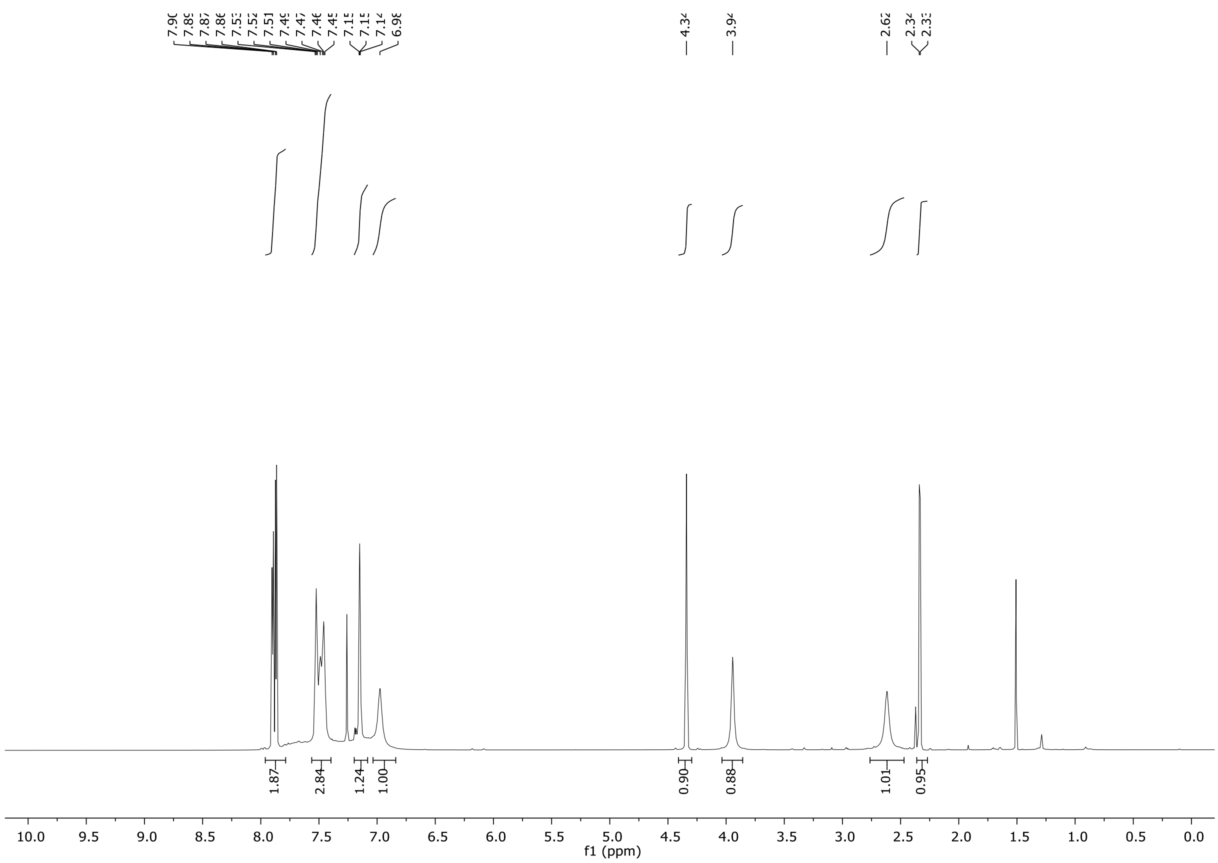

^{1}H NMR (500 MHz) spectrum of **N4d** in $CDCl_3$ measured at 40 °C

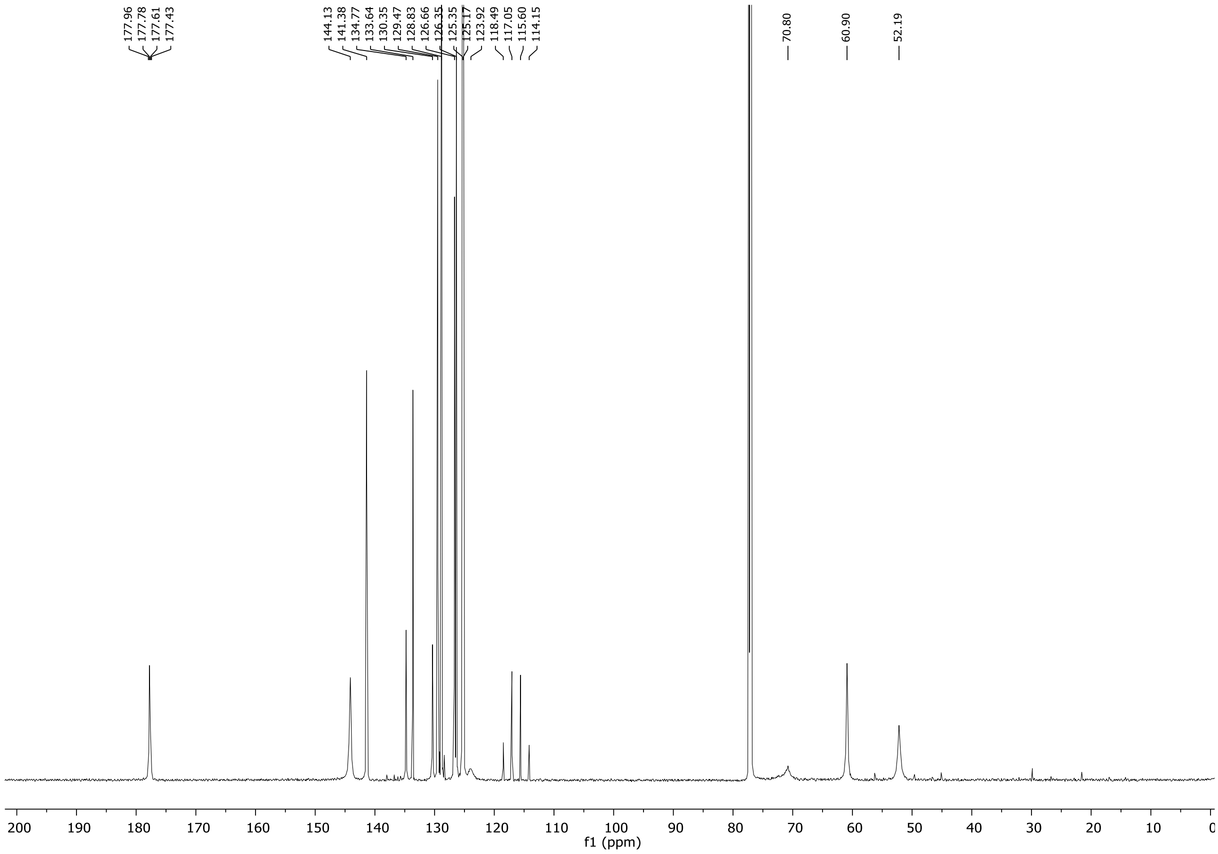

^{13}C NMR (100 MHz) spectrum of **N4d** in $CDCl_3$

Compound **NN4e**

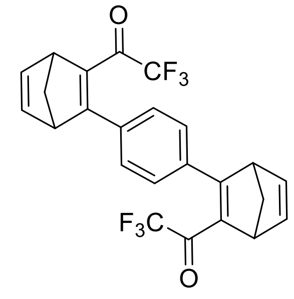

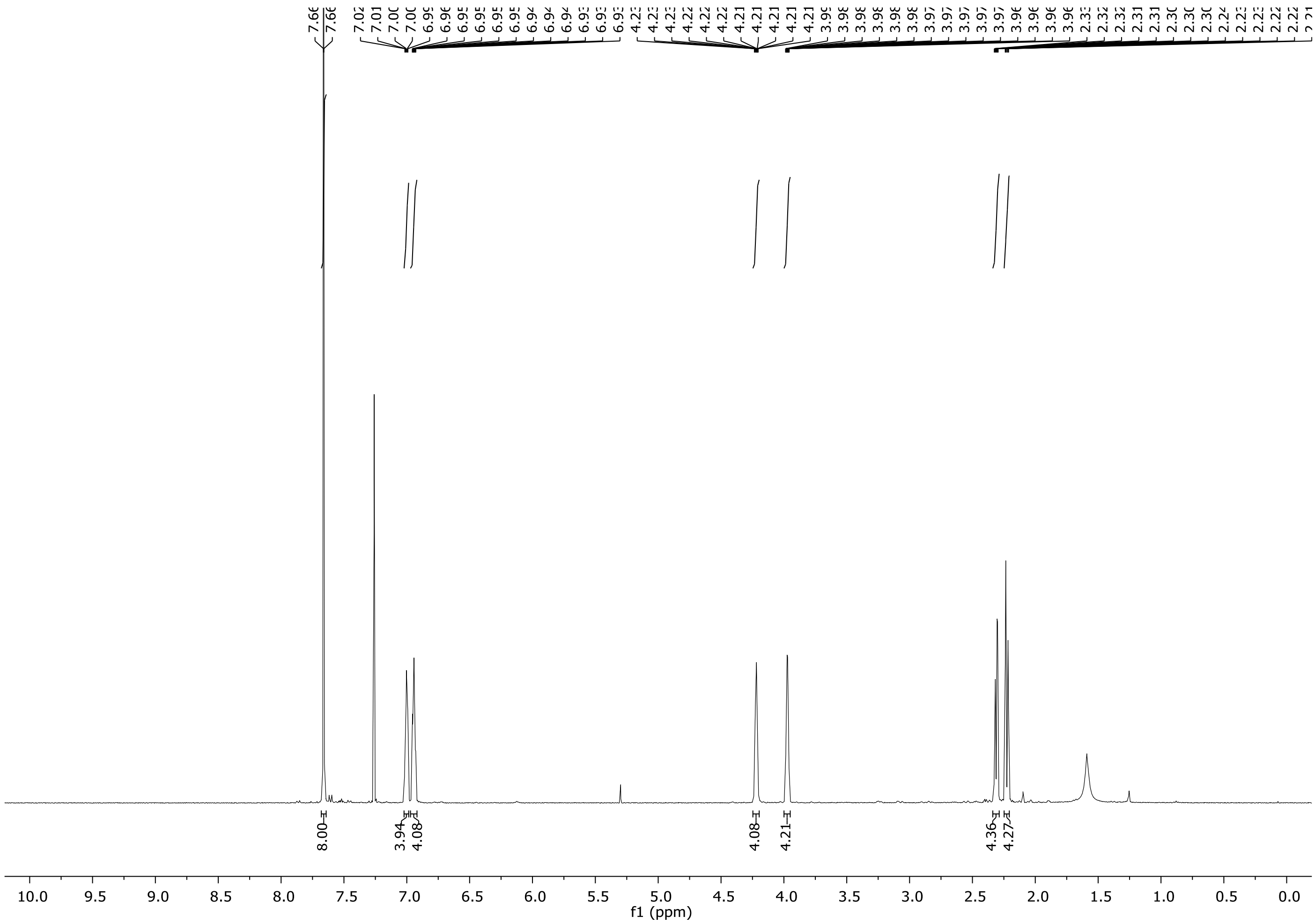

^{1}H NMR (400 MHz) spectrum of **NN4e** in $CDCl_3$

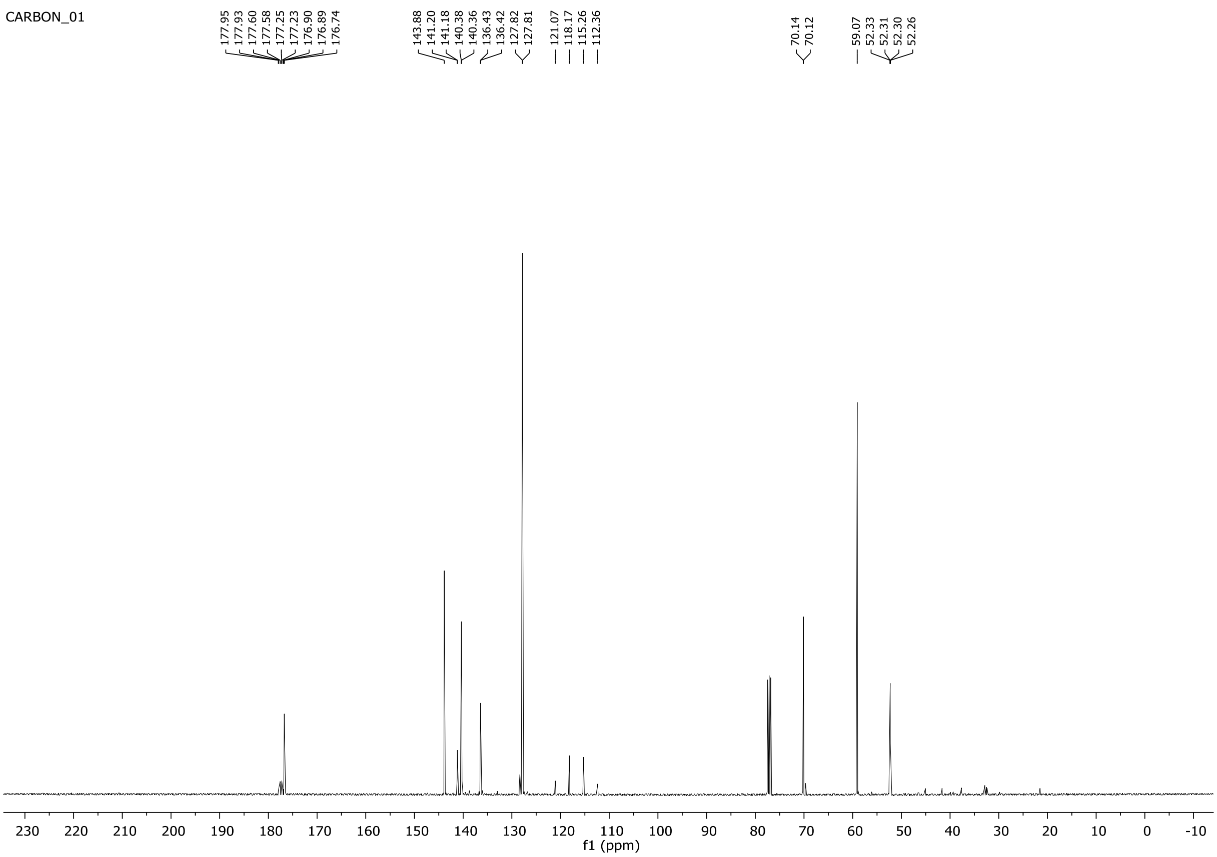

^{13}C NMR (100 MHz) spectrum of **NN4e** in $CDCl_3$

General procedure for the formation of QCs

For the purpose of characterisation, a solution of prerequisite NBD (between 2-20 mg) in either $CDCl_3$ or toluene-d_8 was sparged with argon and subjected to the required light source for such time that no further change in the ^{1}H NMR spectrum was noticed. It was not possible to obtain NMR data for **Q4c** on the account of the short half-life for this metastable.

In addition, neat **Q4b-d**, **QQ4e** and **QQ11** could not be studied by DSC on the account of short half-lives in their neat forms. For the slower back converting QCs, it was possible to obtain IR data by irradiating a film of the neat NBD on an ATR attachment. The obtained data are listed as follows.

Compound **Q4b**

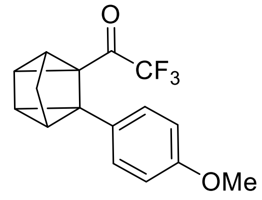

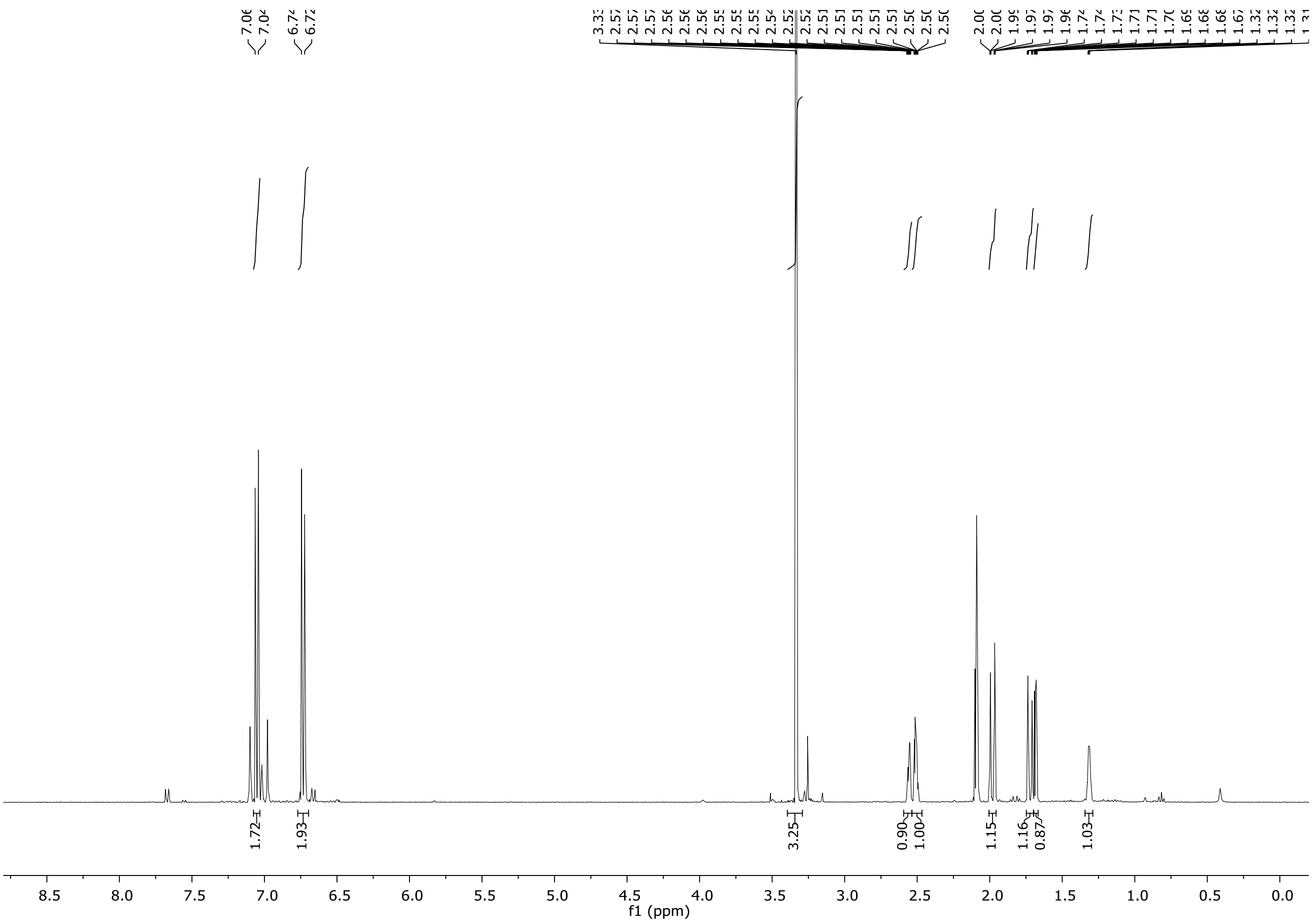

^{1}H NMR (400 MHz) spectrum of **Q4b** in toluene-d_8

2-Trifluoroacetyl-3-(4-methoxyphenyl)quadricyclane (Q4b). ^{1}H NMR (500 MHz, toluene-d_8): $\delta = 7.05$ (d, $J = 8.8$ Hz, 2H), 6.73 (d, $J = 8.8$ Hz, 2H), 3.33 (s, 3H), 2.57–2.54 (m, 1H), 2.52–2.50 (m, 1H), 1.98 (dt, $J = 11.8$, 1.5 Hz, 1H), 1.72 (dt, $J = 11.8$, 1.5 Hz, 1H), 1.68 (dd, $J = 4.8$, 2.5 Hz, 1H), 1.34–1.28 (m, 1H) ppm.

Compound **Q4d**

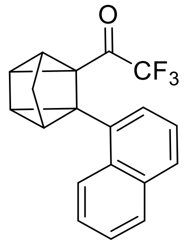

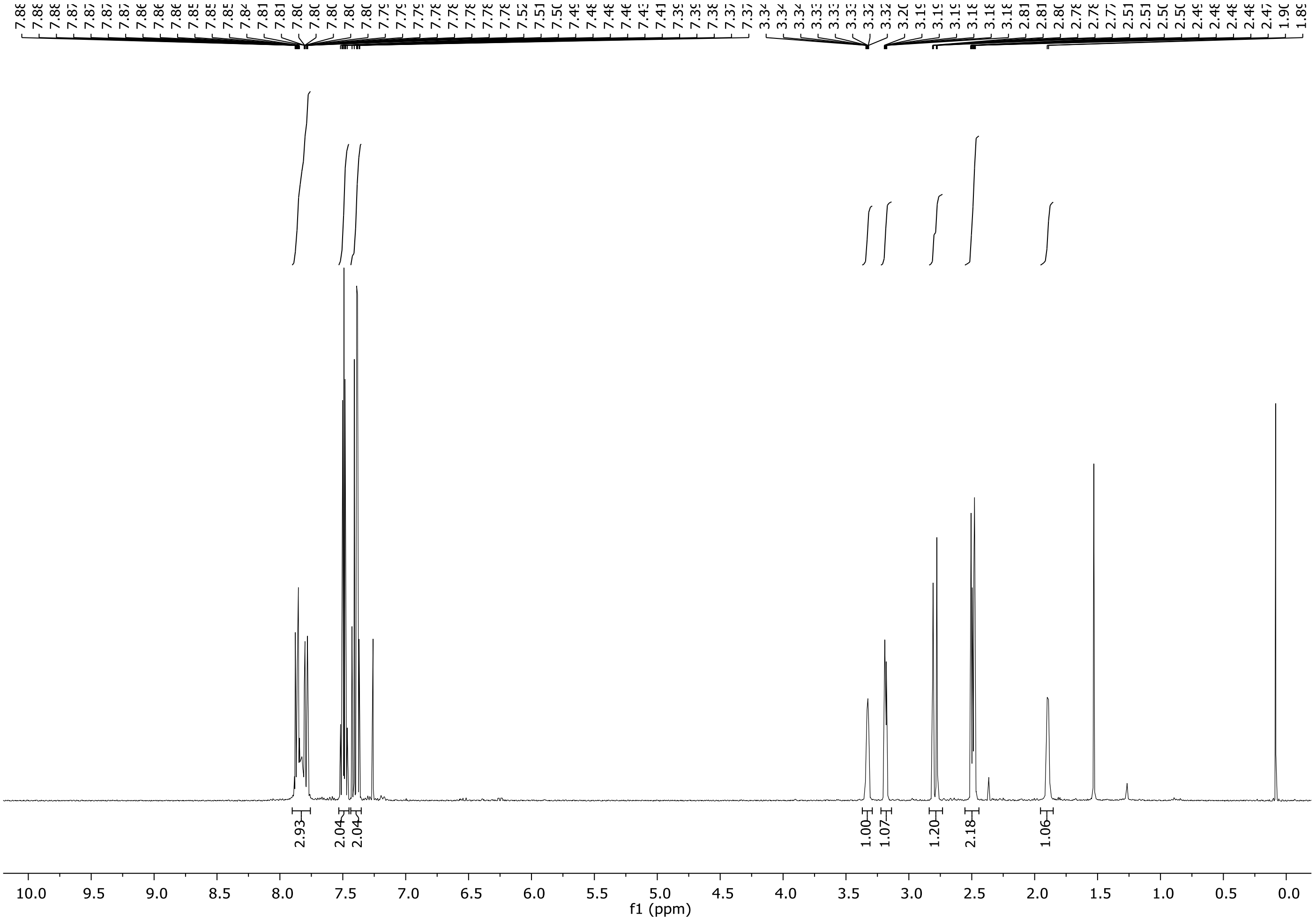

^{1}H NMR (400 MHz) spectrum of **Q4d** in $CDCl_3$

2-Trifluoroacetyl-3-(1-naphthyl)quadricyclane (Q4d). IR: υ_{max} = 3048, 2937, 2865, 1697, 1595, 1509 cm^{-1}. ^{1}H NMR (500 MHz, $CDCl_3$): δ = 7.91–7.77 (m, 3H), 7.52–7.46 (m, 2H), 7.43–7.36 (m, 2H), 3.33 (ddt, J = 5.0, 2.3, 1.4 Hz, 1H), 3.19 (dp, J = 5.0, 1.4 Hz, 1H), 2.79 (dt, J = 12.1, 1.4 Hz, 1H), 2.49 (dt, J = 12.1, 1.4 Hz, 1H), 2.49 (dd, J = 4.6, 2.3 Hz, 1H), 1.90 (br d, J = 4.6 Hz, 1H) ppm. ^{1}H NMR (800 MHz, toluene-d_8): δ = 7.84 (br s, 1H), 7.63 (d, J = 8.2 Hz, 1H), 7.56 (d, J = 8.2 Hz, 1H), 7.32 (ddd, J = 8.2, 7.0, 1.2 Hz, 1H), 7.25 (ddd, J = 8.2, 7.0, 1.2 Hz, 1H), 7.20 (dd, J = 8.2, 7.0 Hz, 1H), 7.11 (dd, J = 7.0, 1.2 Hz, 1H), 2.63 – 2.60 (m, 2H), 2.21 (d, J = 12.2 Hz, 1H), 1.85 (dd, J = 4.8, 2.3 Hz, 1H), 1.84 (dt, J = 12.2, 1.4 Hz, 1H), 1.30 (br s, 1H) ppm. ^{13}C NMR (200 MHz, toluene-d_8): δ = 184.45 (q, J = 33.5 Hz), 134.94, 134.05, 132.64, 128.92, 128.47, 127.06, 126.08, 125.83, 125.27, 116.42 (q, J = 293.3 Hz), 41.21, 40.05, 35.95, 32.71, 32.46, 30.97, 19.79 ppm (1C masked).

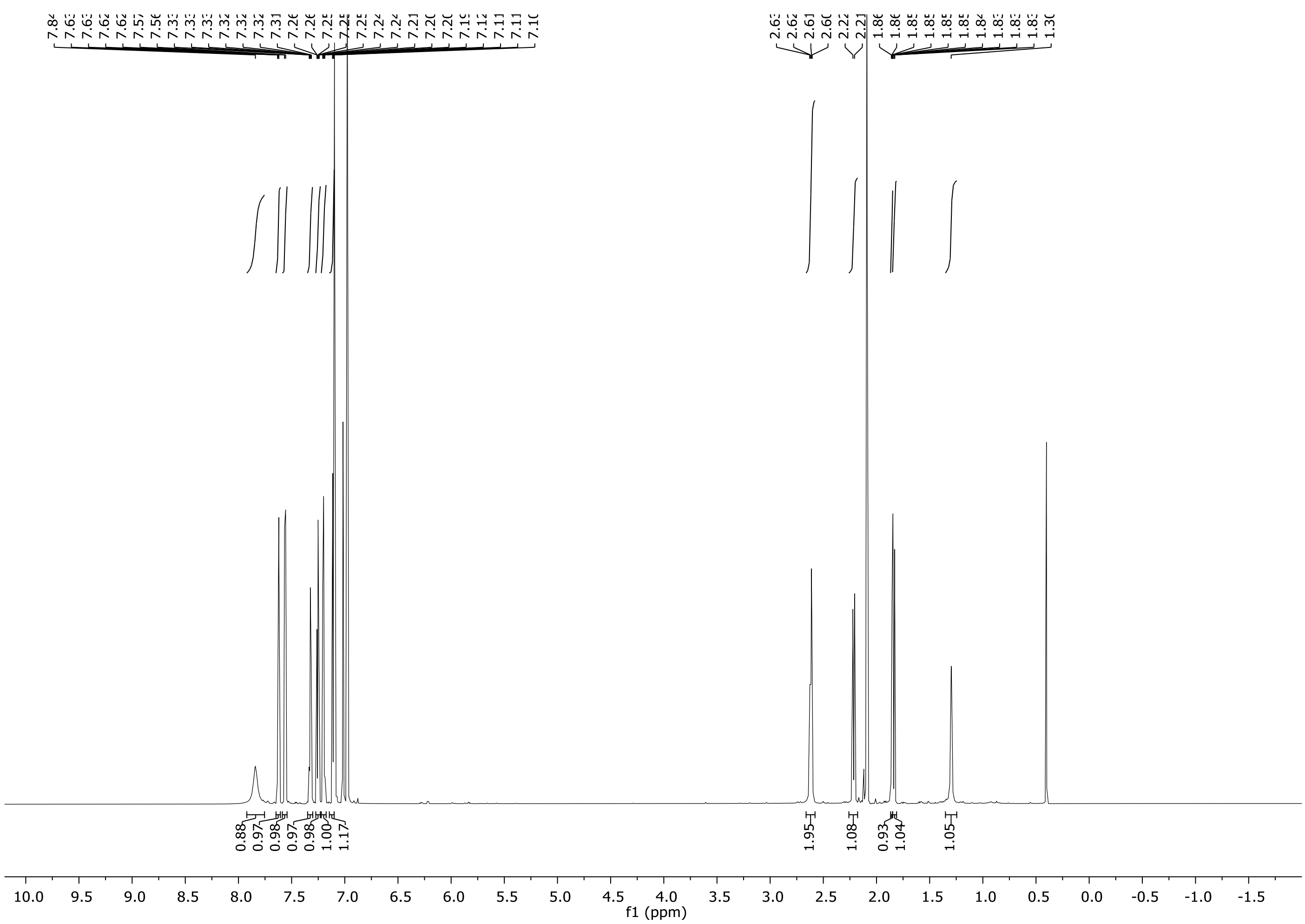

^{1}H NMR (800 MHz) spectrum of **Q4d** in toluene-d_8

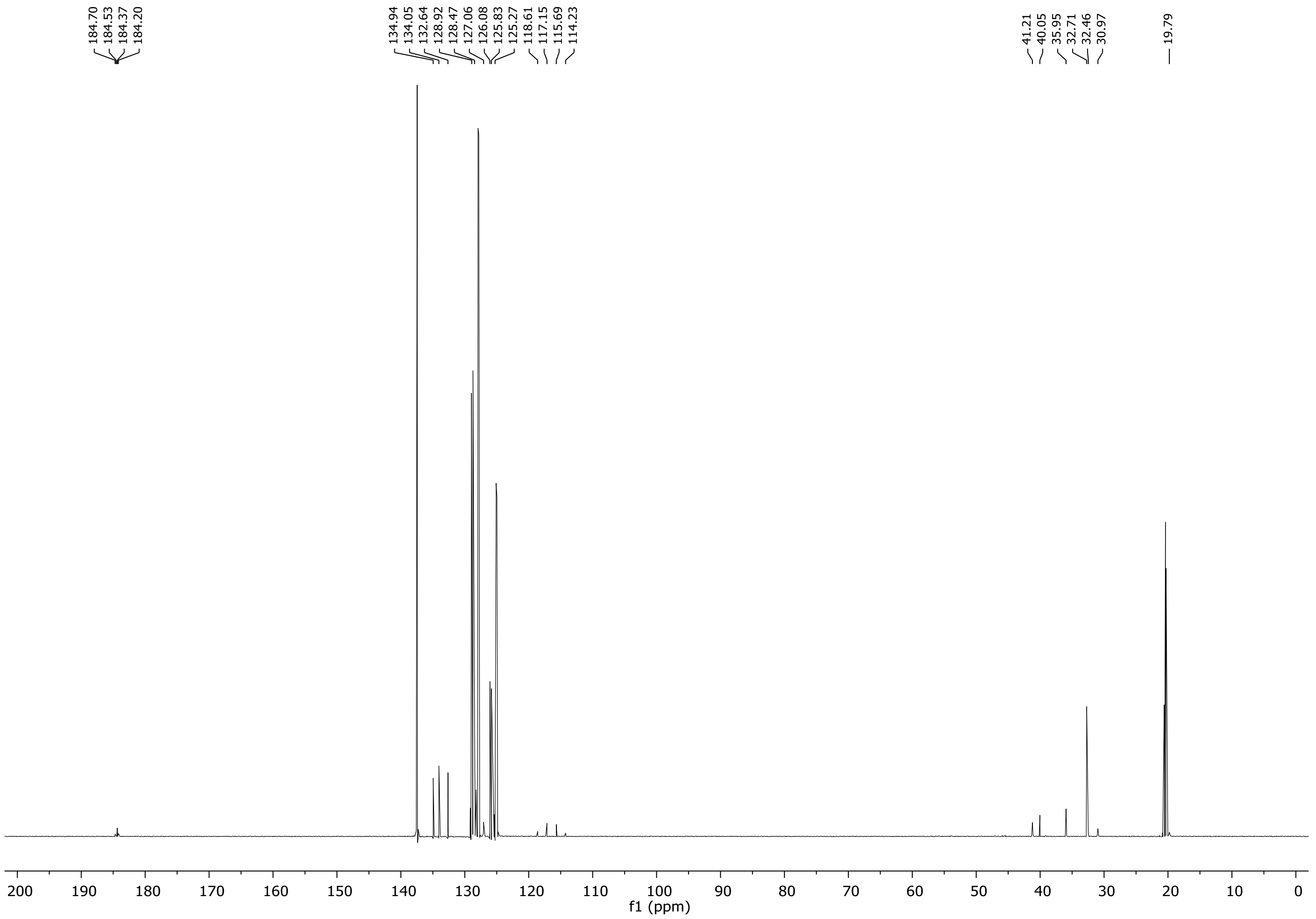

^{13}C NMR (201 MHz) spectrum of **Q4d** in toluene-d_8

Compound **QQ4e**

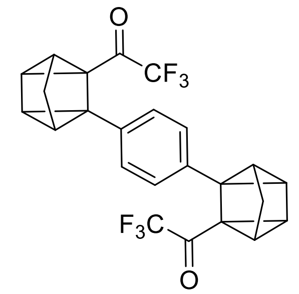

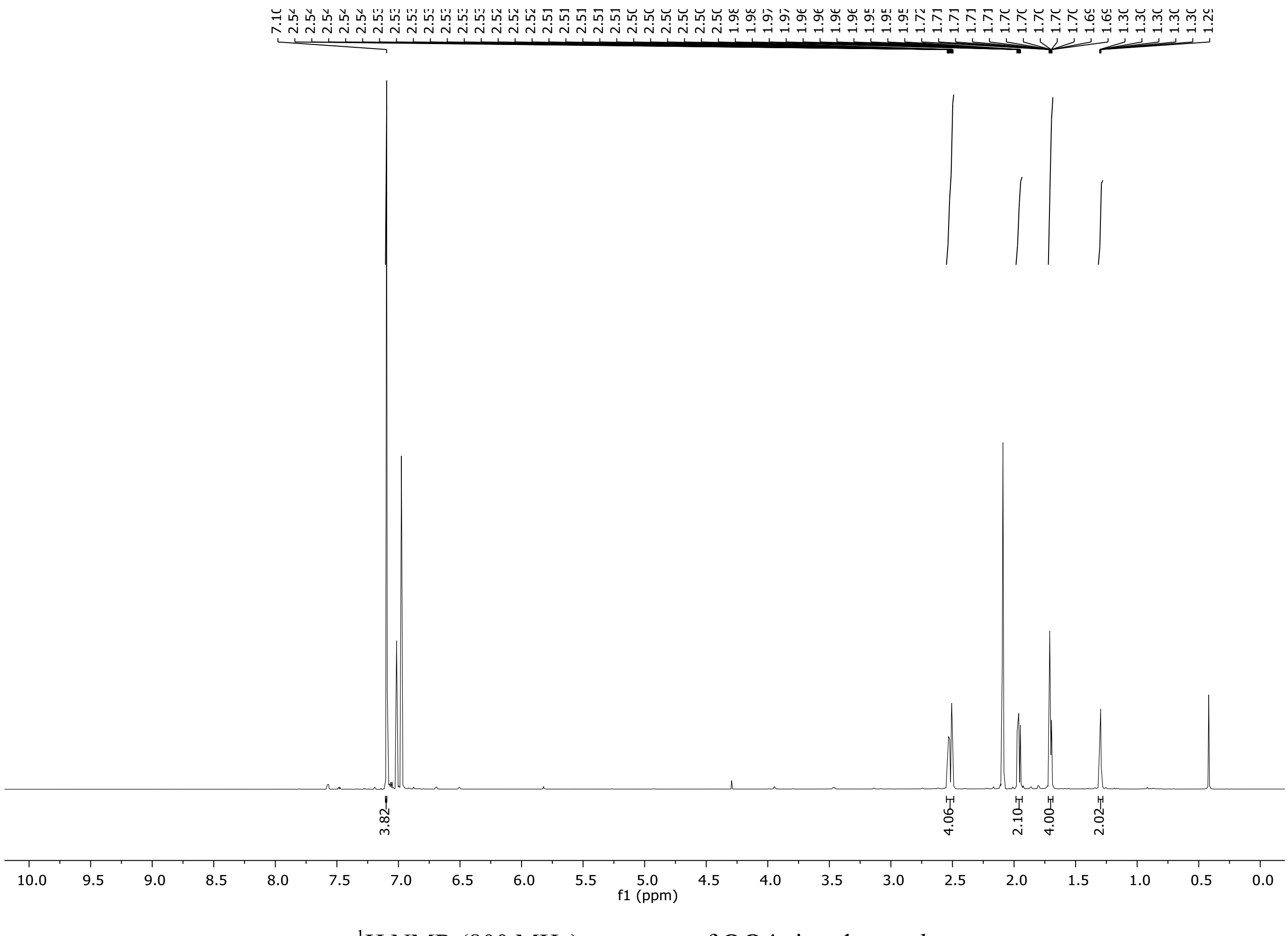

^{1}H NMR (800 MHz) spectrum of **QQ4e** in toluene-d_8

Compound QQ4e. IR: υ_{max} = 1696, 1524 cm^{-1}. ^{1}H NMR (800 MHz, toluene-d_8): δ = 7.10 (s, 4H), 2.55–2.49 (m, 4H), 1.96 (ddt, J = 12.1, 9.3, 1.4 Hz, 2H), 1.73–1.68 (m, 4H), 1.30–1.29 (m, 2H) ppm.

UV-Vis absorbance and kinetic studies in solution

Compound **N4b**

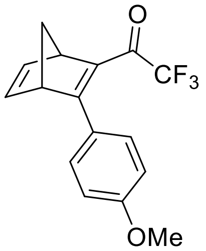

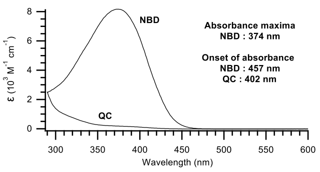

Absorbance spectrum

Extinction coefficient for the NBD calculated as the average of three solutions (8064.4, 8166.2, 8275.6) gives 8168.9 $M^{-1}cm^{-1}$.

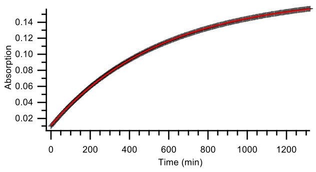

Increase of NBD at 25 °C

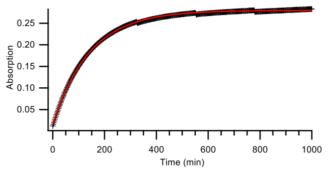

Increase of NBD at 40 °C

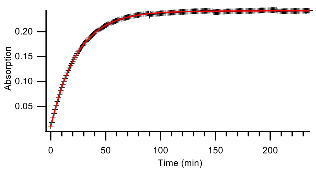

Increase of NBD at 60.0 °C

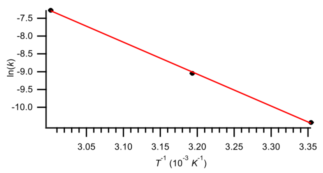

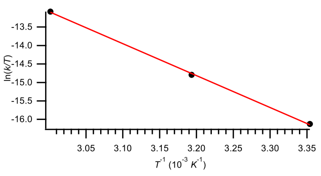

Above
Arrhenius plot giving the values
$A = 3.2 \cdot 10^{8}\ s^{-1}$, $E_a = 74445\ J\ mol^{-1}$

Left
Eyring plot giving the values
$\Delta H = 71.8$ kJ/mol $\Delta S = -90.9\ J\ mol^{-1}\ K^{-1}$

Compound **N4c**

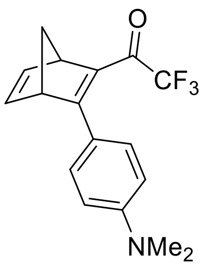

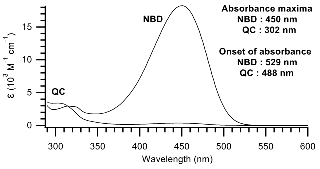

Absorbance spectrum

Extinction coefficient for the NBD calculated as the average of three solutions (18115, 18215, 18373) gives 18234 $M^{-1}cm^{-1}$.

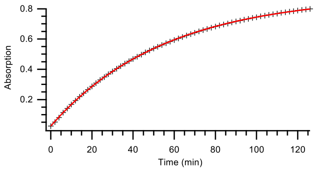

Increase of NBD at 25 °C

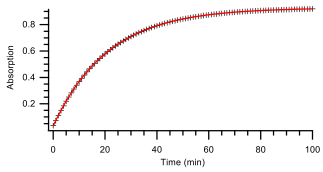

Increase of NBD at 35 °C

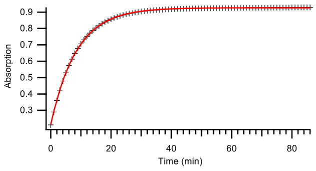

Increase of NBD at 60.0 °C

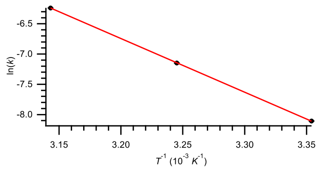

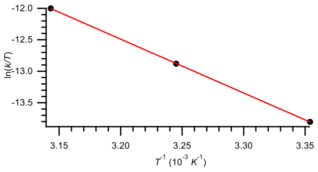

Above
Arrhenius plot giving the values
$A = 2.46 \cdot 10^9$ s^{-1}, $E_a = 73703$ J mol^{-1}

Left
Eyring plot giving the values
$\Delta H = 71.1$ kJ/mol $\Delta S = -73.7$ J mol^{-1} K^{-1}

Compound **N4d**

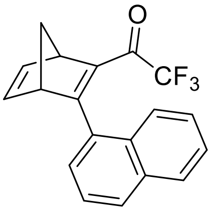

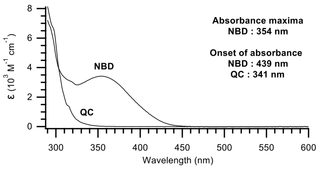

Absorbance spectrum

Extinction coefficient for the NBD calculated as the average of three solutions (3419, 3411, 3409) gives 3413 $M^{-1}cm^{-1}$.

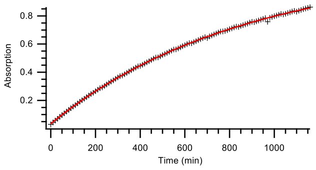

Increase of NBD at 75 °C

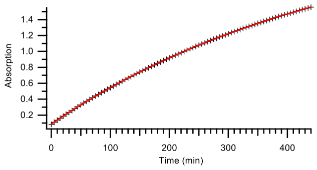

Increase of NBD at 80 °C

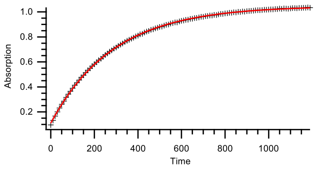

Increase of NBD at 85.0 °C

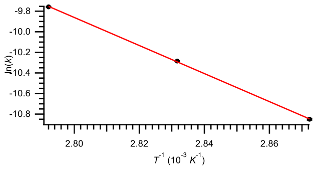

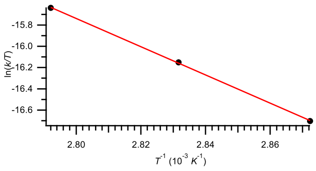

Above
Arrhenius plot giving the values
$A = 2.06 \cdot 10^{12}$ s^{-1}, $E_a = 113467$ J mol^{-1}

Left
Eyring plot giving the values
$\Delta H = 110.5$ kJ/mol $\Delta S = -18.9$ J mol^{-1} K^{-1}

Compound **NN4e**

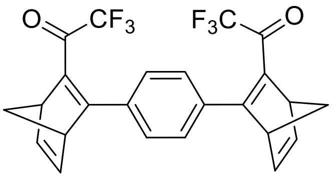

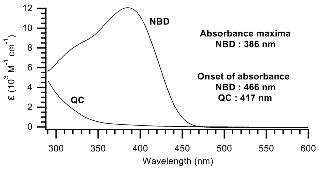

Absorbance spectrum

Extinction coefficient for the NBD calculated as the average of three solutions (12101, 12102, 12178) gives 12127 $M^{-1}cm^{-1}$.

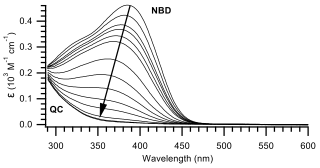

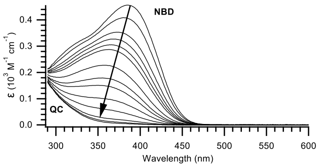

Irradiated at 405 nm

Irradiated at 455 nm

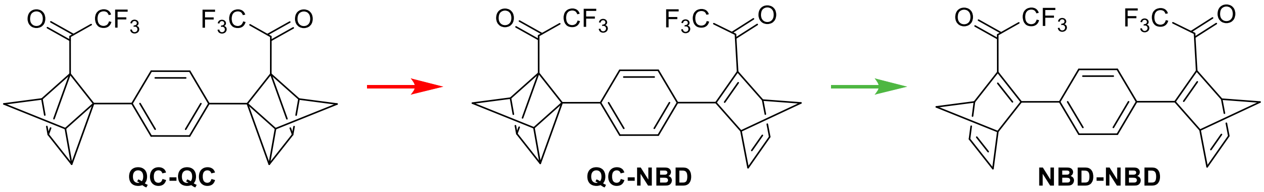

As this molecule has to different rates for the back conversion. The conversion from **QC-QC** to **QC-NBD** is shown in red. The conversion of **QC-NBD** to **NBD-NBD** is shown in green. This is being rationalised by the change in the absorbance maxima, for the first conversion from **QC-QC** to **NBD-NBD**, the maximum of the curve is unchanged, while for the last conversion **QC-NBD** to **NBD-NBD**, λ_{max} is redshifted.

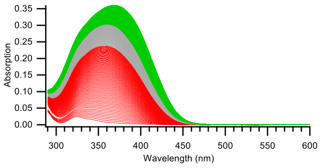

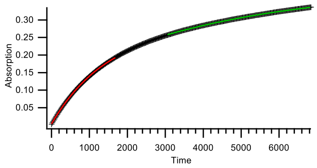

Increase of NBD at 25 °C

Increase of NBD at 25 °C

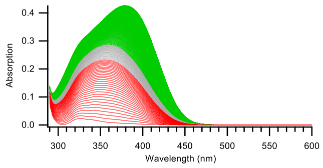

Increase of NBD at 50 °C

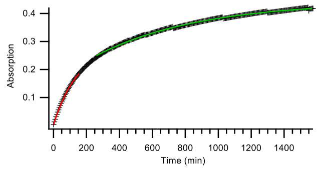

Increase of NBD at 25 °C

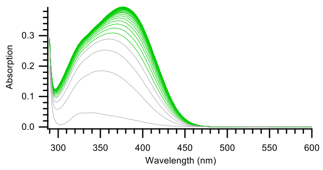

Increase of NBD at 50 °C

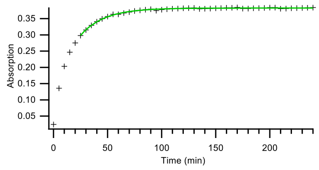

Increase of NBD at 50 °C

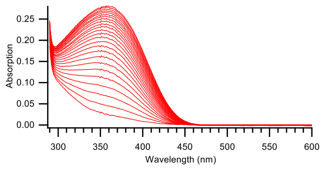

Increase of NBD at 70 °C

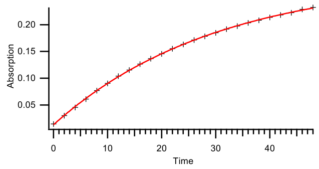

Increase of NBD at 70 °C

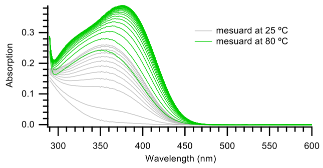

Increase of NBD at 70 °C

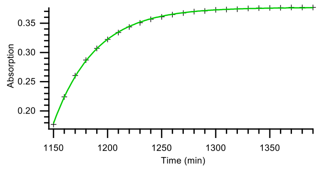

Increase of NBD at 70 °C

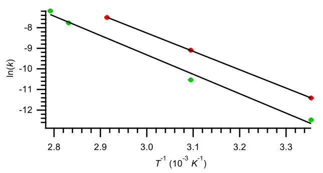

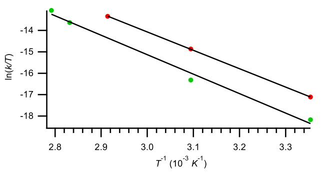

Arrhenius plot giving the values

QC-QC to QC-NBD
A = 9.97 · 10^7 s^{-1}, E_a = 73938 J mol^{-1}

QC-NBD to NBD-NBD
A = 1.62 · 10^8 s^{-1}, E_a = 78231.6 J mol^{-1}

Eyring plot giving the values
QC-QC to QC-NBD
ΔH = 71.3 kJ/mol ΔS = -100.7 J mol^{-1} K^{-1}

QC-NBD to NBD-NBD
ΔH = 75.5 kJ/mol ΔS = -96.8 J mol^{-1} K^{-1}

NMR studies of photo-conversions of **NN4e**

In order to confirm the multimode switching of th dimer, **NN4e** was irradiated on an NMR scale in toluene-d_8.

Compound **NN4e**

The compound was irradiated to full conversion at 405 nm, and the left to back convert.

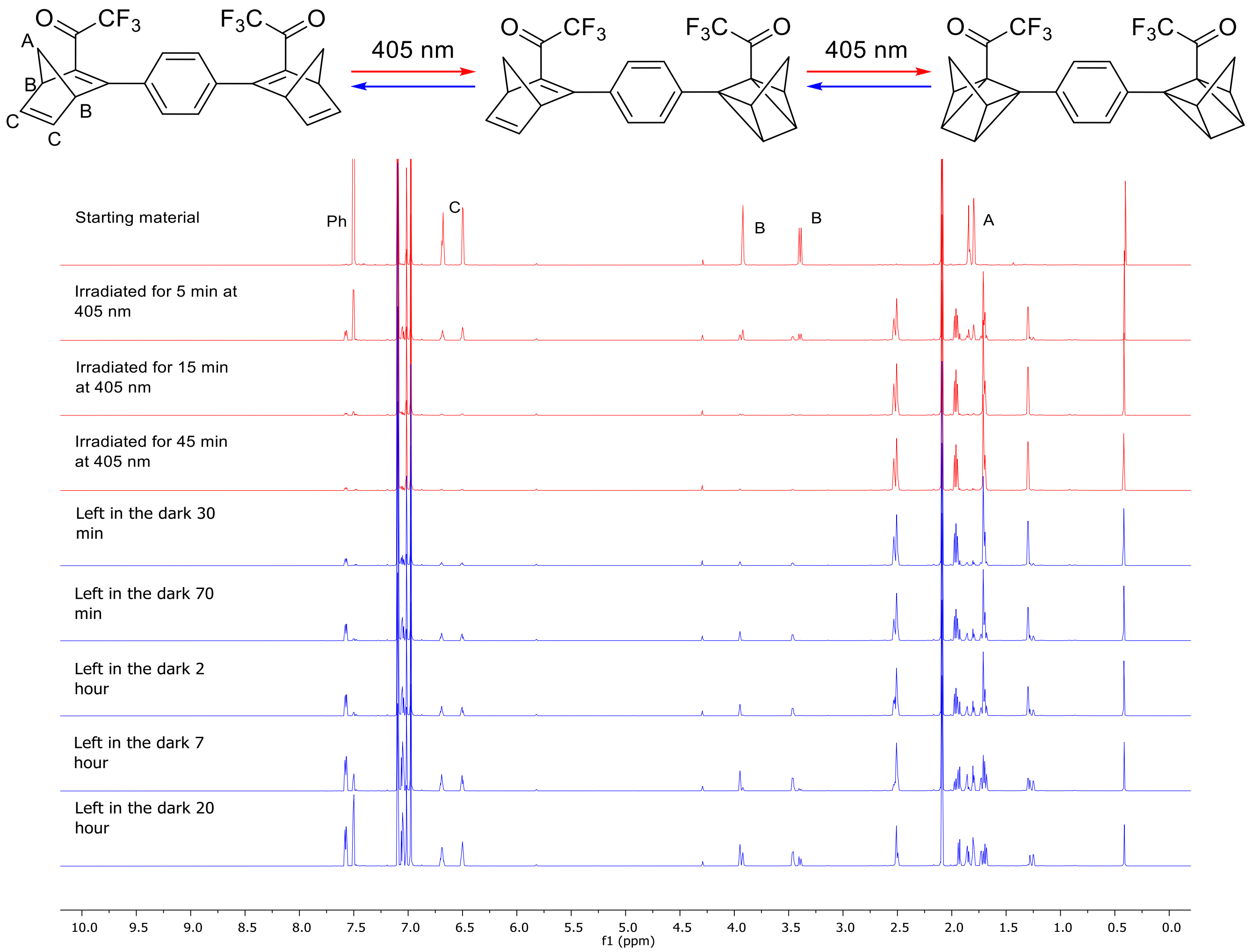

^{1}H NMR (800 MHz) spectra of **NN4e** firstly subjected to irradiation with a 405 nm light source and monitoring the back reaction in toluene-d_8

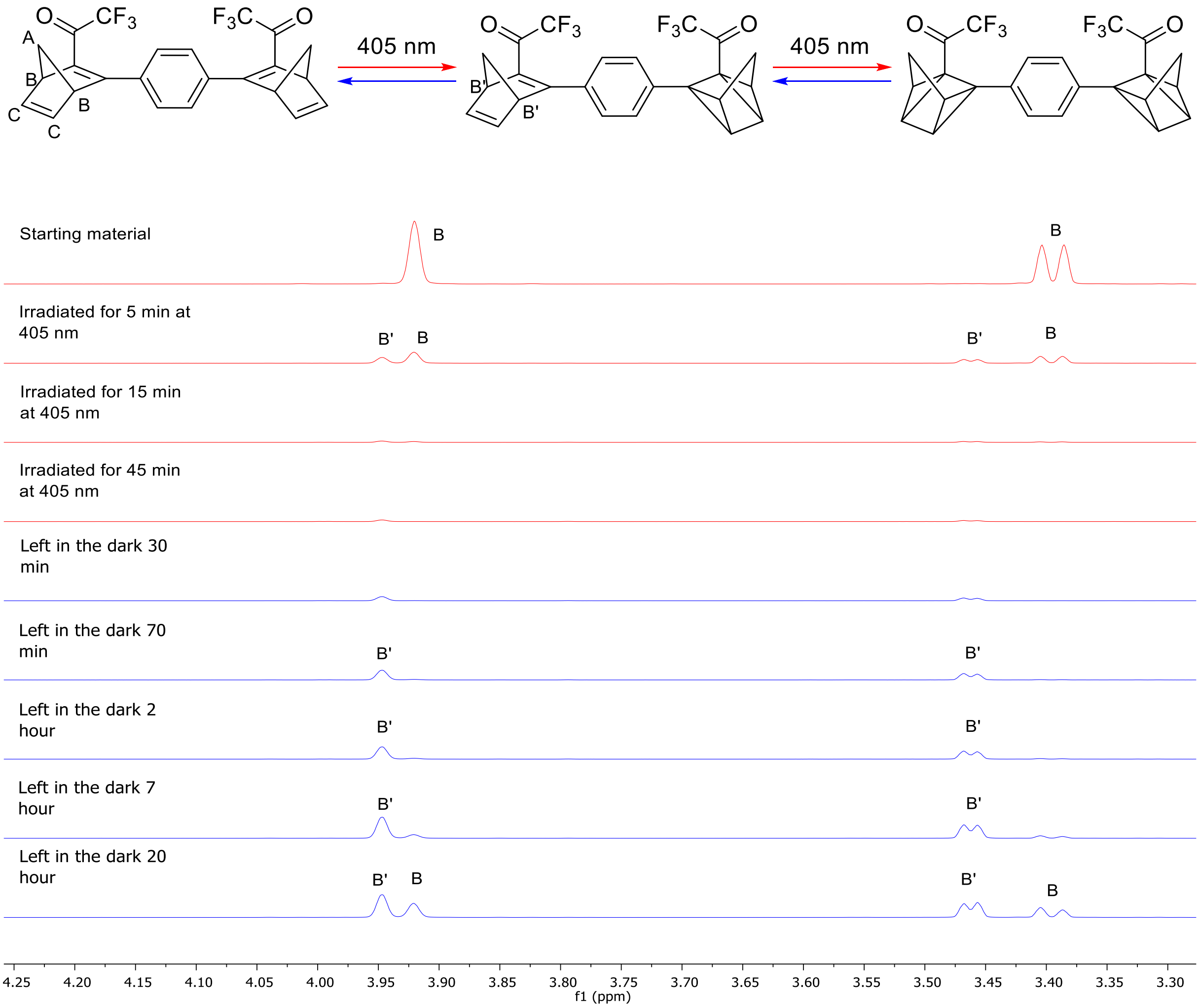

^{1}H NMR (800 MHz) spectra of **NN4e** firstly subjected to irradiation with a 405 nm light source and monitoring the back reaction in toluene-d_8 zoomed in

When examining the peaks belonging to the brigdehead proton it is clear that there is no sequential swiching as both B and B' are present. As for the conversion back to the NBD-NBD it appears that QC-NBD (**QQ4e→NQ4e**) is formed faster than the the NBD-NBD (**NQ4e→NN4e**).

Photoisomerisation quantum yields for **N4b-d**, **NN4e**.

Photon flux measurements

340 nm LED
Photon flux: 9.327 x 10^{-9} mol s^{-1}
Used with: **N4d**, **NN4e**

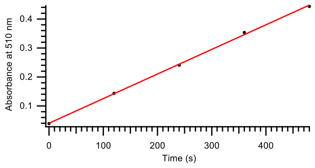

405 nm LED
Photon flux: 1.318 x 10^{-7} mol s^{-1}
Used with: **N4b**

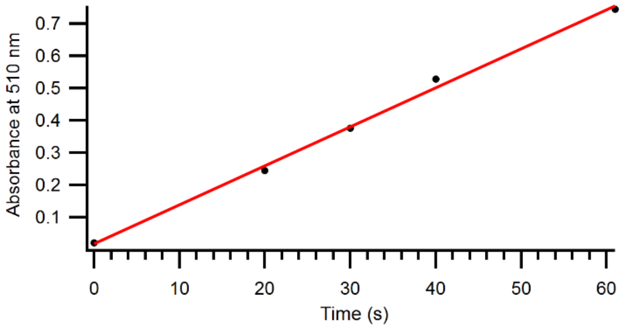

405 nm LED
Photon flux: 1.0766 x 10^{-7} mol s^{-1}
Used with: **NN4e, NN11**

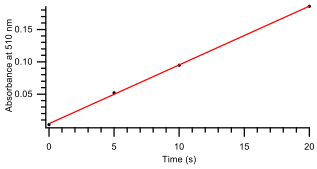

455 nm LED
Photon flux: 2.2160 x 10^{-7} mol s^{-1}
Used with: **N4c**

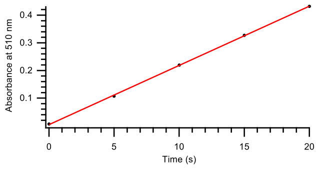

Compound **N4b**

Mesurad at 405 nm

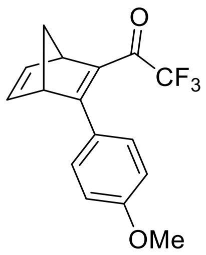

ϕ_1 = 67.69 %
ϕ_2 = 68.35 %
$\phi_{average}$ = 68.02 %

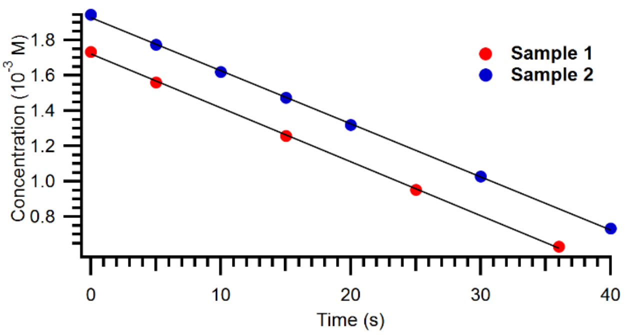

Compound **N4c**

Measured at 455 nm

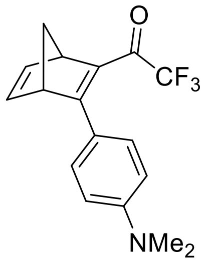

ϕ_1 = 54.05 %
ϕ_2 = 53.65 %
$\phi_{average}$ = 53.85 %

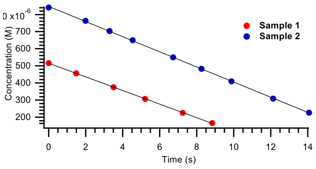

This measurement was performed at 15 °C where the half life of the QC is 108 minutes. At the same time the measurement was done as fast as possible, only irradiating for a short time, and only scanning the absorbance from 575 nm to 440 nm, in order to measure an absorbance under 1, and so to ensure that the absorbance values are higher than 2 at 455 nm where the sample is irradiated.

Compound **N4d**

Measured at 340 nm

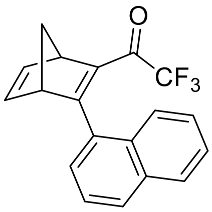

ϕ_1 = 45.93 %
ϕ_2 = 46.97 %
$\phi_{average}$ = 46.45

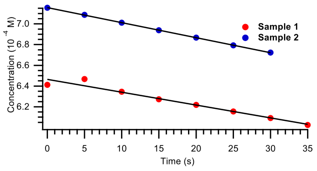

Compound **NN4e**

Measured at 340 nm

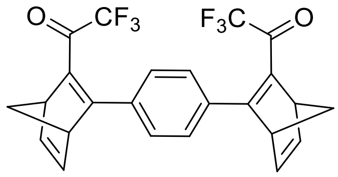

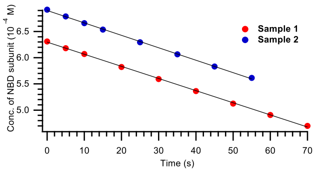

Calculated pr NBD sub unit

$\phi_1 = 76.37\ \%$

$\phi_2 = 80.09\ \%$

$\phi_{average} = 78.41$

Measured at 405 nm

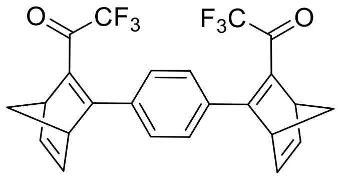

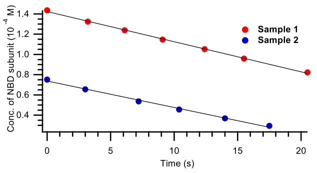

Calculated per NBD sub unit

$\phi_1 = 78.67\ \%$

$\phi_2 = 74.27\ \%$

$\phi_{average} = 76.70$

Incorporation of NBD into a polymer

Incorporation N4b in a polymer film. A polymer solution in CH_2Cl_2 was mixed with a solution of **N4b** and poured into a petri dish, which was left on a 25 °C heating plate for the solvent to slowly evaporate overnight. The sample was then dried under high vacuum to ensure the evaporation of all volatiles.

Polymer	Conc. of polymer sol.	Volume of polymer sol.	NBD	Conc. of NBD sol.	Volume of NBD solution	Loading of NBD in composit
PMMA	100 mg/mL	10 mL	**N4b**	10 mg/mL	0.1 mL	0.10 wt %
PS	100 mg/mL	10 mL	**N4b**	10 mg/mL	0.1 mL	0.10 wt %
PC	100 mg/mL	10 mL	**N4b**	10 mg/mL	0.1 mL	0.10 wt %
PVDC	100 mg/mL	10 mL	**N4b**	10 mg/mL	0.1 mL	0.10 wt %

Incorporation of N4c into a polymer film. A polymer solution in CH_2Cl_2 was mixed with a solution of **N4c** and degassed before being poured into a petri dish, which was left on a 25 °C heating plate for the solvent to slowly evaporate overnight. The sample was then dried under high vacuum to ensure the evaporation of the CH_2Cl_2.

Polymer	Conc. of polymer sol.	Volume of polymer sol.	NBD	Conc. of NBD sol.	Volume of NBD solution	Loading of NBD in composit
PS	100 mg/mL	1 mL	**N4c**	10 mg/mL	0.05 mL	0.50 wt %
PS	100 mg/mL	5 mL	**N4c**	10 mg/mL	0.05 mL	0.10 wt %
PS	100 mg/mL	10 mL	**N4c**	10 mg/mL	0.05 mL	0.05 wt %
PS	200 mg/mL	20 mL	**N4c**	10 mg/mL	0.01 mL	0.005 wt %

Incorporation NBDs into PS for DSC measurements. Around a 10 wt% loading of NBD in PS was dissolved in CH_2Cl_2 and, as this was a very small amount, the mixed poured into a short NMR tube or a cup of tin foil and left on a 25 °C heating plate for the solvent to slowly evaporate overnight forming a small pellet. The sample was then dried under high vacuum to ensure the evaporation of the CH_2Cl_2. In order to get the precise loading of the NBD, a sample of the NBD@PS was weighed and dissolved in 3 mL of toluene. The absorbance of the solution was measured by UV-Vis spectroscopy to determine the concentration of NBD in solution. This provides the amount of NBD in the weighed sample, and thereby the loading of the sample.

NBD	ε (Abs_{max})	Abs_{max}	Conc. (mol/L)	n (mol)	Mw (g/mol)	Mass NBD (mg)	Mass of composit (mg)	Loading (wt%)
**N4b**@PS	8169	0.66437	$8.13 \cdot 10^{-5}$	$2.44 \cdot 10^{-7}$	294.09	0.0718	0.85	8.44
**N4d**@PS	3413	0.15737	$4.61 \cdot 10^{-5}$	$1.38 \cdot 10^{-7}$	314.31	0.0435	0.34	12.79
**NN4e**@PS	12127	1.1725	$9.67 \cdot 10^{-5}$	$2.90 \cdot 10^{-7}$	450.38	0.131	1.09	11.98

UV-Vis absorbance and kinetic study of **N4b** in polymers

N4b@PMMA

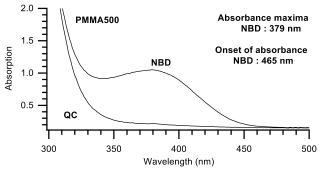

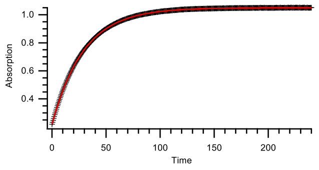

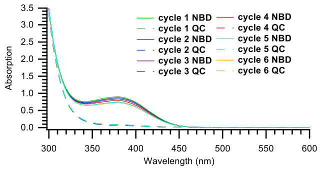

Increase of NBD at ambient temperature

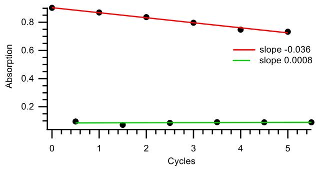

N4b@PC

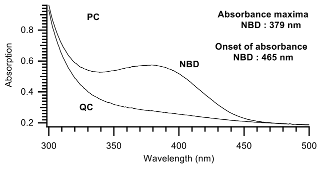

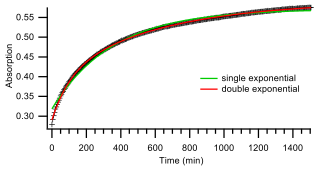

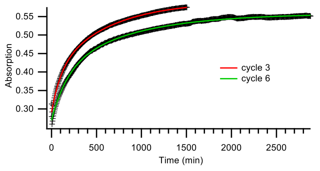

Increase of NBD at ambient tempratrue

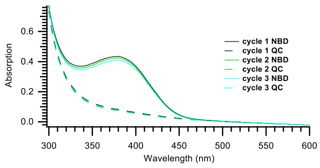

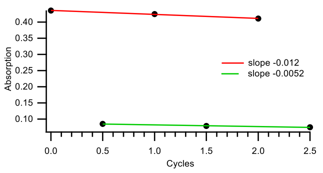

N4b@PS

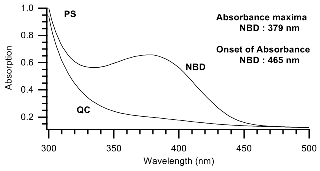

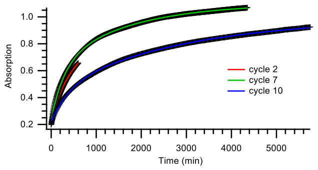

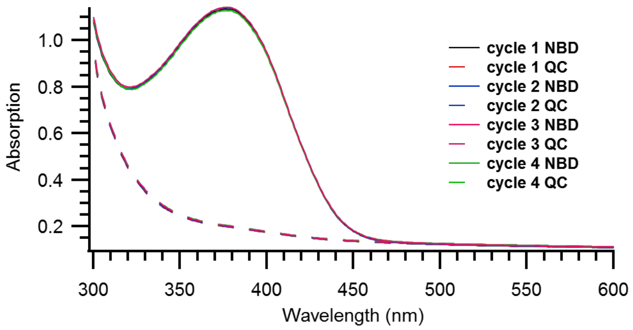

Increase of NBD at ambient tempratrue

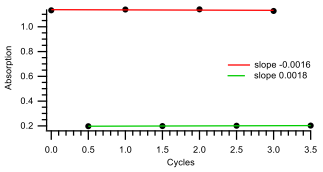

N4b@PVDC

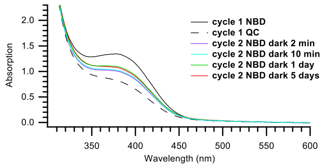

UV-Vis absorbance and kinetic study of **N4c** in PS

N4c@PS

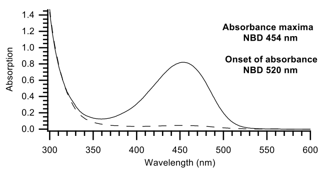

Weight percent of 0.005 % **N4c** in PS

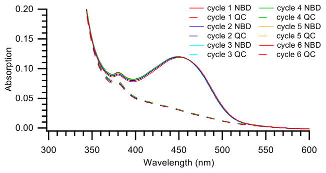

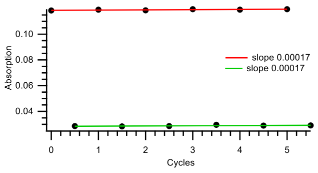

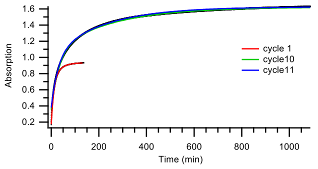

Weight percent of 0.05 % **N4c** in PS

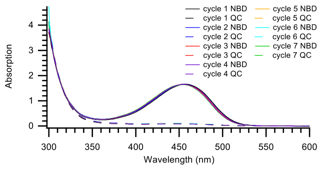

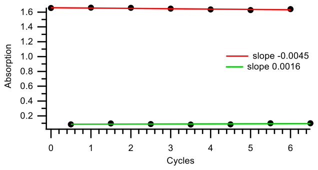

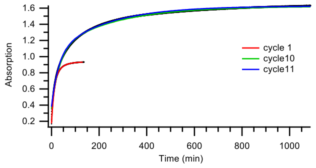

Weight percent of 0.10 % **N4c** in PS

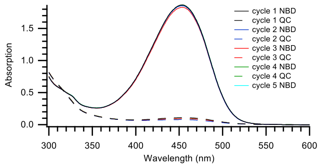

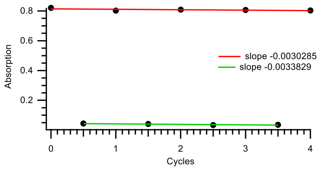

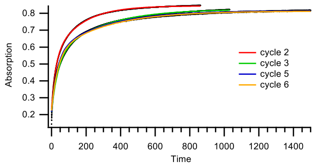

Weight percent of 0.50 % **N4c** in PS

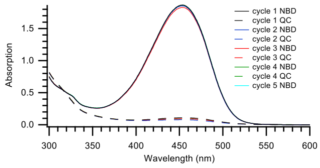

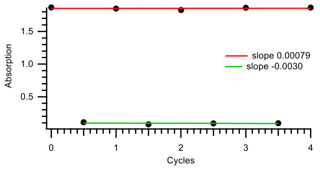

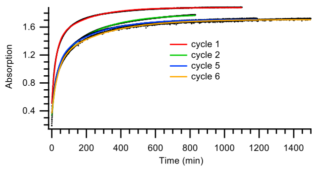

Heat release measurements for QCs in polymers

The NBD@PS composite was weighed and placed in a DSC pan. This was then irradiated with a solar simulator until the color had disapeared and the polymer sample appeared transparent. The pan was sealed and the heat release of the sample was measured in the DSC. After the measurement the lid was removed and the sample was carefully transfered with tweezers to a new pan. The sample was weighed again, as small pieces could break off in the transfer to a new DSC pan. The process of using the solar simulator in conjunction with DSC was repeated two to three times.

Before irradiation

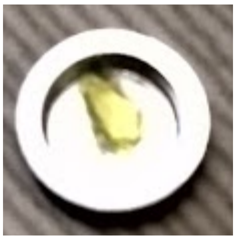

After Irradiation

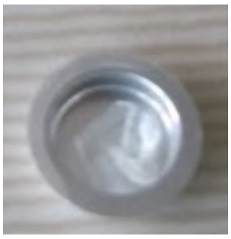

Pictures of DSC sampels of **N4d**@PS before and after irradition

Compound **N4b**

Sample 1

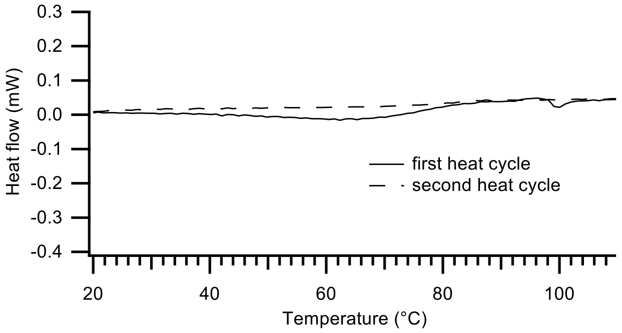

Heating rate 2 °C/min
Heated from 15°C - 110 °C
Amount 1.16 mg
Integration area: 26.98 – 91.40 °C

Heat release: 34.42 kJ/kg

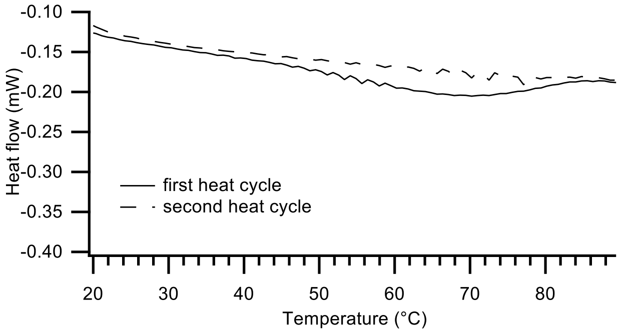

Heating rate 2 °C/min
Heated from 15°C - 90 °C
Amount 1.06 mg
Integration area: 32.09 – 85.94 °C

Heat release: 24.97 kJ/kg

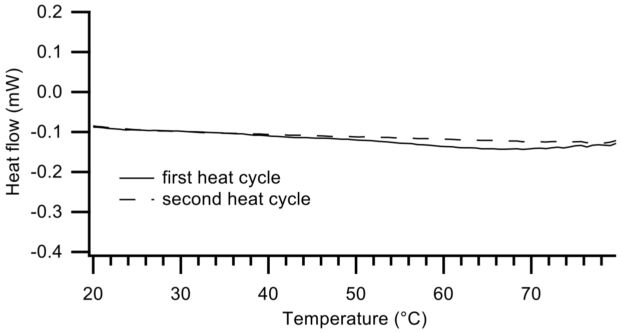

Heating rate 2 °C/min
Heated from 15°C - 80 °C
Amount 1.06 mg
Integration area: 35.06 – 78.27 °C

Heat release: 12.17 kJ/kg

Sample 2

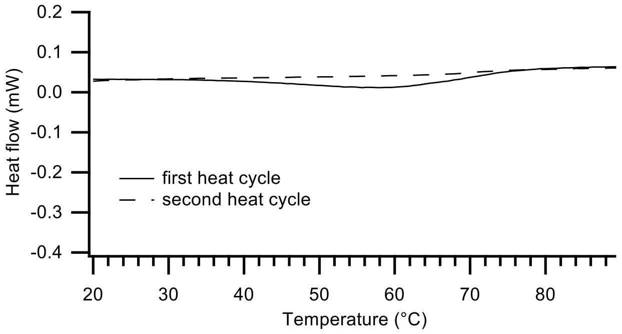

Heating rate 2 °C/min
Heated from 15°C - 90 °C
Amount 1.51 mg
Integration area: 27.61 – 83.31 °C

Heat release: 27.74 kJ/kg

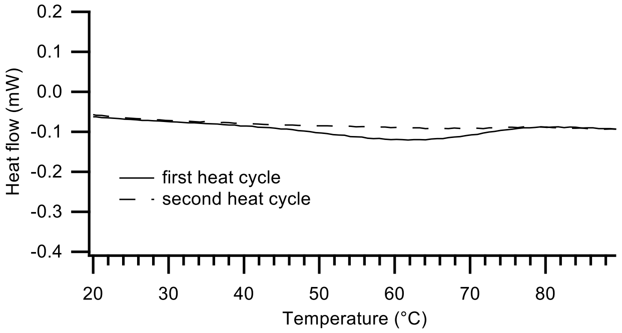

Heating rate 2 °C/min
Heated from 15°C - 90 °C
Amount 1.47 mg
Integration area: 28.24 – 81.25 °C

Heat release: 28.89 kJ/kg

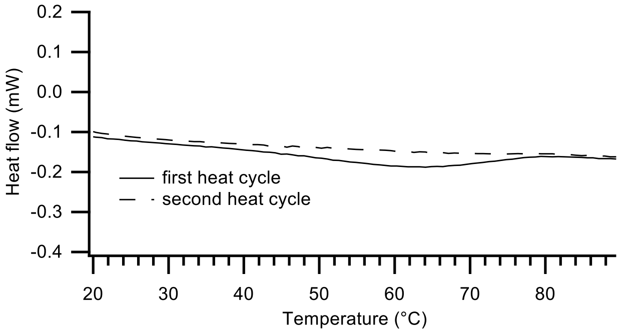

Heating rate 2 °C/min
Heated from 15°C - 90 °C
Amount 1.47 mg
Integration area: 30.83 – 82.25 °C

Heat release: 29.41 kJ/kg

Sample 3

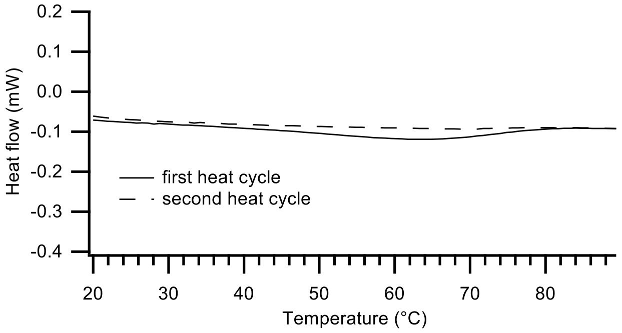

Heating rate 2 °C/min
Heated from 15°C - 90 °C
Amount 1.44 mg
Integration area: 20.86 – 85.30 °C

Heat release: 32.07 kJ/kg

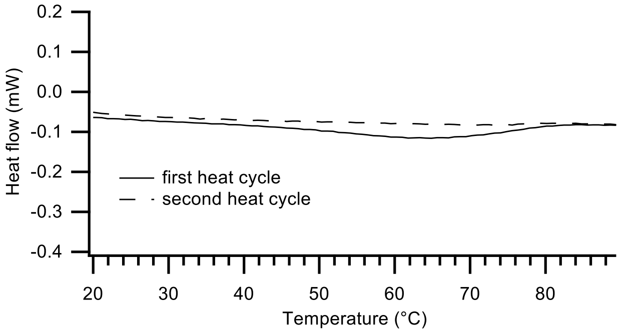

Heating rate 2 °C/min
Heated from 15°C - 90 °C
Amount 1.40 mg
Integration area: 28.06 – 84.50 °C

Heat release: 30.65 kJ/kg

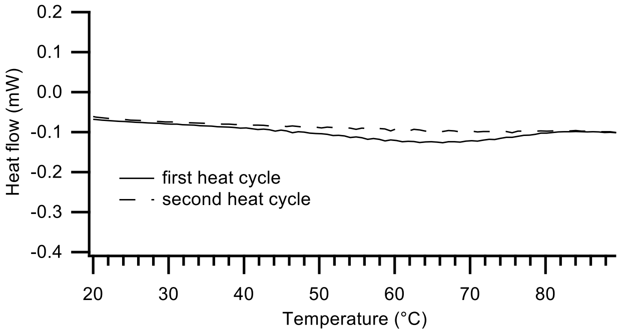

Heating rate 2 °C/min
Heated from 15°C - 90 °C
Amount 1.40 mg
Integration area: 30.76 – 83.87 °C

Heat release: 27.16 kJ/kg

Sample 4

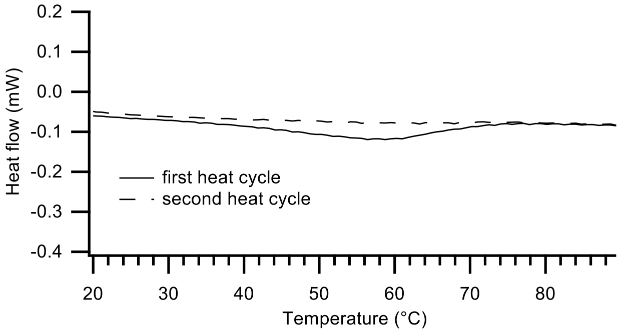

Heating rate 2 °C/min
Heated from 15°C - 90 °C
Amount 1.47 mg
Integration area: 30.04 – 77.12 °C

Heat release: 28.39 kJ/kg

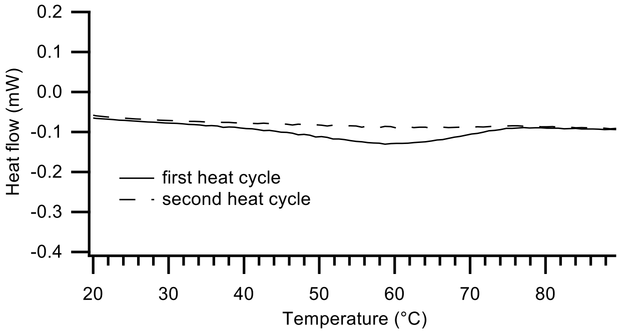

Heating rate 2 °C/min
Heated from 15°C - 90 °C
Amount 1.47 mg
Integration area: 29.59 – 79.64 °C

Heat release: 31.20 kJ/kg

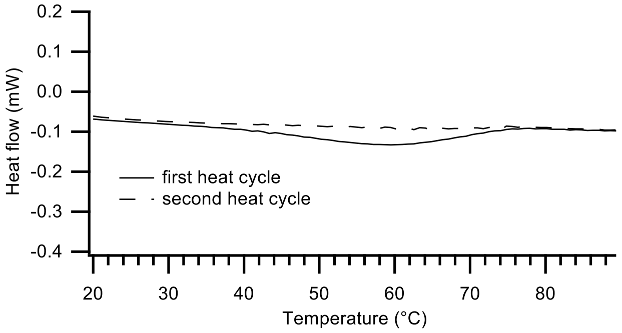

Heating rate 2 °C/min
Heated from 15°C - 90 °C
Amount 1.42 mg
Integration area: 29.77 – 79.46 °C

Heat release: 32.40 kJ/kg

Compound **N4d**

Sample 1

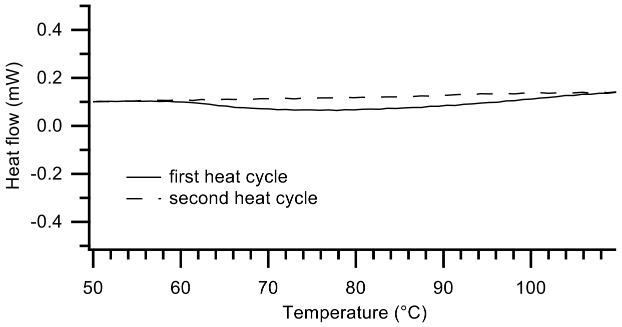

Heating rate 2 °C/min
Heated from 15°C - 110 °C
Amount 0.95 mg
Integration area: 49.36 – 109.36 °C

Heat release: 50.39 kJ/kg

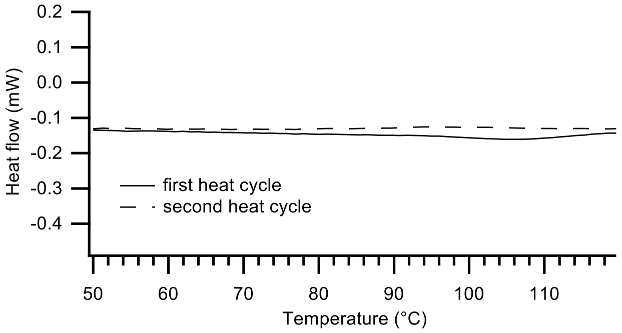

Heating rate 2 °C/min
Heated from 15°C - 120 °C
Amount 0.91 mg
Integration area: 49.16 – 118.85 °C

Heat release: 18.00 kJ/kg

Sample 2

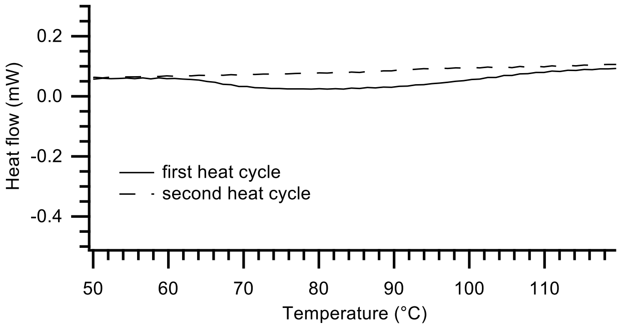

Heating rate 2 °C/min
Heated from 15°C - 120 °C
Amount 1.00 mg
Integration area: 56.45 – 117.47 °C

Heat release: 50.53 kJ/kg

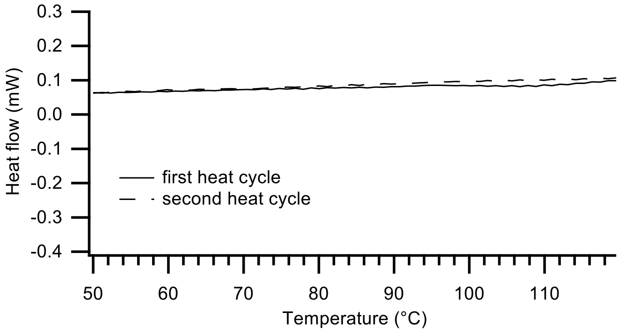

Heating rate 2 °C/min
Heated from 15°C - 120 °C
Amount 1.00 mg
Integration area: 36.31 – 116.84 °C

Heat release: 1.66 kJ/kg

Sample 3

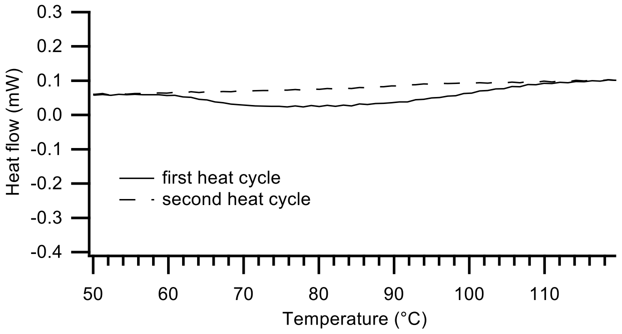

Heating rate 2 °C/min
Heated from 15°C - 120 °C
Amount 0.92 mg
Integration area: 53.76 – 117.73 °C

Heat release: 50.34 kJ/kg

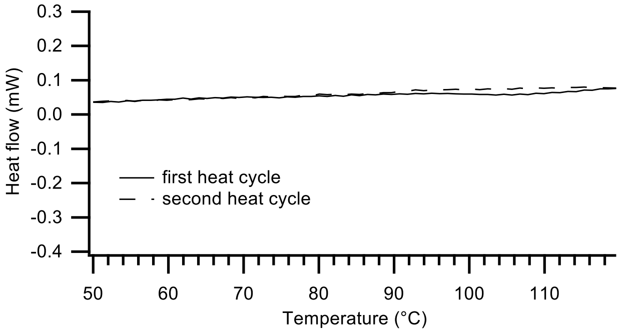

Heating rate 2 °C/min
Heated from 15°C - 120 °C
Amount 0.80 mg
Integration area: 84.20 – 118.35 °C

Heat release: 4.80 kJ/kg

Compound **NN4e**

Sample 1

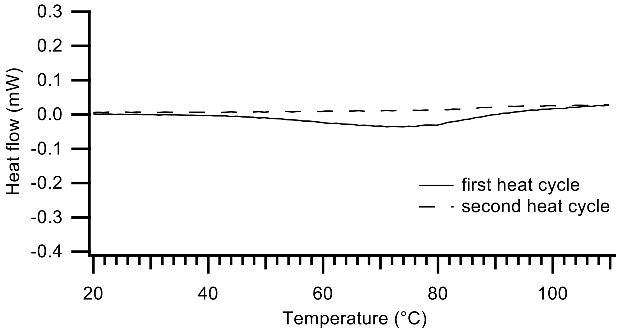

Heating rate 2 °C/min
Heated from 15°C - 110 °C
Amount 1.44 mg
Integration area: 34.62 – 105.43 °C

Heat release: 48.97 kJ/kg

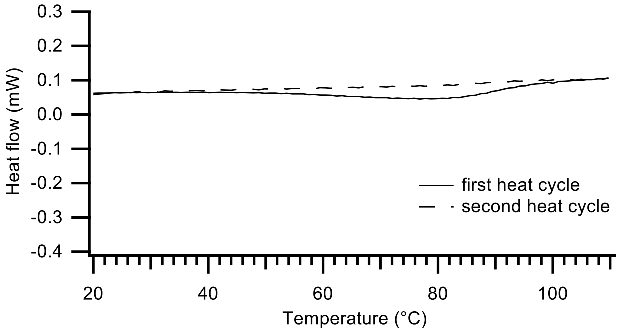

Heating rate 2 °C/min
Heated from 15°C - 110 °C
Amount 1.39 mg
Integration area: 32.26 – 106.29 °C

Heat release: 43.35 kJ/kg

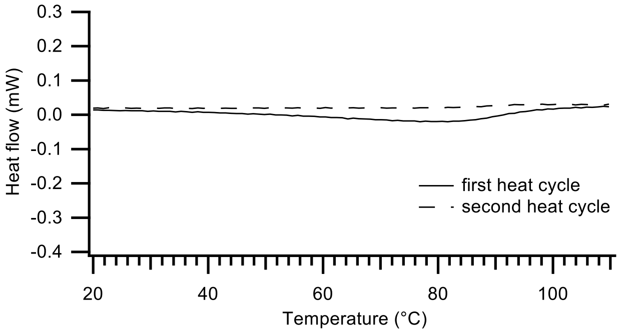

Heating rate 2 °C/min
Heated from 15°C - 110 °C
Amount 1.39 mg
Integration area: 32.58 – 106.92 °C

Heat release: 38.96 kJ/kg

Sample 2

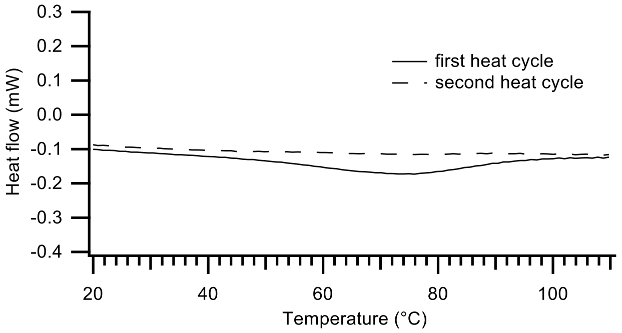

Heating rate 2 °C/min
Heated from 15°C - 110 °C
Amount 1.17 mg
Integration area: 26.62 – 100.96 °C

Heat release: 55.21 kJ/kg

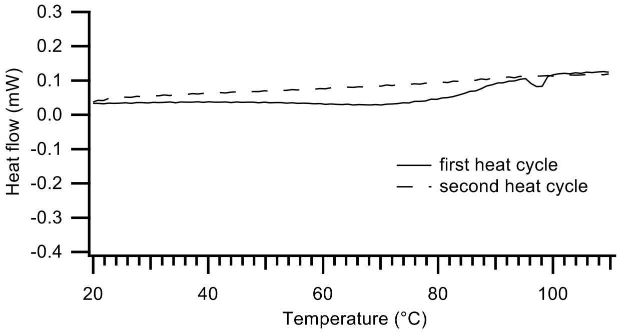

Heating rate 2 °C/min
Heated from 15°C - 110 °C
Amount 1.15 mg
Integration area: 31.87 – 95.39 °C

Heat release: 47.96 kJ/kg

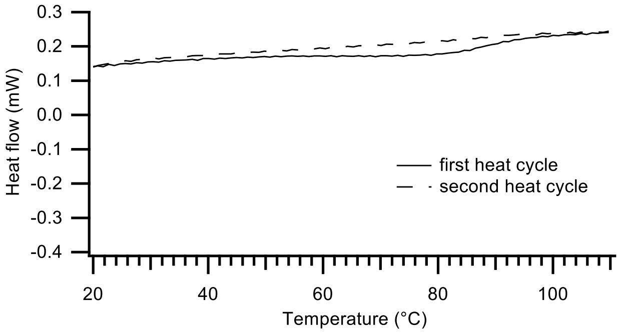

Heating rate 2 °C/min
Heated from 15°C - 110 °C
Amount 1.16 mg
Integration area: 36.50 – 102.37 °C

Heat release: 29.92 kJ/kg

Sample 3

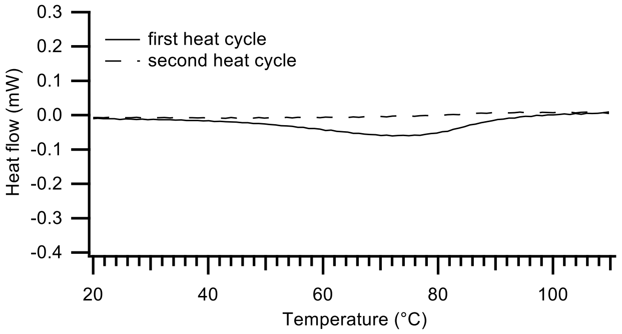

Heating rate 2 °C/min
Heated from 15°C - 110 °C
Amount 1.14 mg
Integration area: 32.11 – 100.89 °C

Heat release: 51.08 kJ/kg

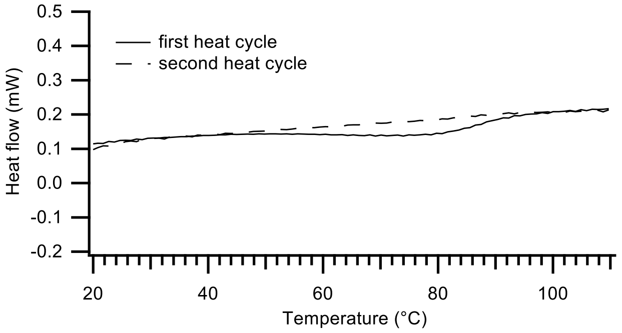

Heating rate 2 °C/min
Heated from 15°C - 110 °C
Amount 1.11 mg
Integration area: 35.87 – 95.94 °C

Heat release: 34.37 kJ/kg

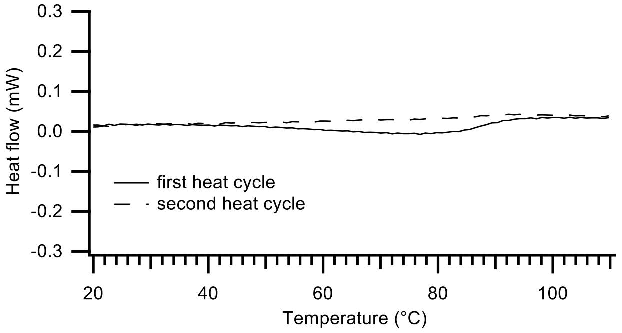

Heating rate 2 °C/min
Heated from 15°C - 110 °C
Amount 1.11 mg
Integration area: 35.09 – 98.22 °C

Heat release: 32.90 kJ/kg

Theoretical maximum energy storage

Assuming all incident light is absorbed by N4b, N4d and NN4e, the maximum energy storage efficiency can be calculated individually from equation (1)[1]:

$$\eta_{\mathrm{MOST}} = \frac{\int_0^{\lambda_{\mathrm{onset}}} \frac{E_{\mathrm{AM\,1.5}}(\lambda) \cdot \phi_{\mathrm{iso}} \cdot \Delta H_{stor}}{h\nu \cdot N_{\mathrm{A}}} \cdot d\lambda}{\int E_{\mathrm{AM\,1.5}}(\lambda) \cdot d\lambda} \cdot 100\ \% \qquad (1)$$

Where $E_{\mathrm{AM\,1.5}}(\lambda)$ corresponds to the spectral irradiance (energy current density) in J s^{-1} m^{-2} nm^{-1}; h represents to the Plank constant in J s; v and N_{A} are the frequency of incoming light in s^{-1} and Avogadro's constant respectively. The input of the equation and maximum energy storage efficiency for N4b, N4d and NN4e are shown in table 1:

Table 1: Inputs for equation (1) and theoretical maximum energy storage efficiency for N4b, N4d and NN4e.

	ϕ_{iso}	$\Delta H^{\ddagger}_{\mathrm{storage}}$ (kJ mol^{-1})	Onset of absorption (nm)	MAX efficiency
**N4b**	0.68	105	457	**2.86%**
**N4d**	0.46	110	439	**1.50%**
**NN4e**	0.77/2*	216	466	**3.78%**

*: 2 photons can only charge one NN4e to QQ4e

1 Z. Wang, J. Udmark, K. Börjesson, R. Rodrigues, A. Roffey, M. Abrahamsson, M. B. Nielsen and K. Moth-Poulsen, *ChemSusChem*, **2017**, 10, 3049–3055.
